# Supplementary material for: Potential G-quadruplexes and i-Motifs in the SARS-CoV-2
Source: PLoS One. 2021 Jun 8;16(6):e0250654. doi: 10.1371/journal.pone.0250654 (PMC8186786; doi:10.1371/journal.pone.0250654)
Supplement: S1 File — (PDF) [file pone.0250654.s001.pdf]

Supplementary material for

Potential G-quadruplexes and i-Motifs in the SARS-CoV-2

Efres Belmonte-Reche [1*], Israel Serrano-Chacón [2], Carlos Gonzalez [2], Juan Gallo [1], Manuel Bañobre-López [1].

[1] Advanced (magnetic) Theranostic Nanostructures Lab, INL-International Iberian Nanotechnology Laboratory, Av. Mestre José Veiga, 4715-330 Braga, Portugal.

[2] Instituto de Química Física'Rocasolano', CSIC, 28006 Madrid, Spain.

* To whom correspondence should be addressed; Phone, +351-253140112;
e-mail: efres.belmonte@inl.int.

Contents table

1. G4-iM Grinder, methodological scheme and upgrades S3
G4-iM Grinder algorithm S3
Methodological scheme S6
Upgrades S7
2. Genomes used S8
3. Results S9
SARS-CoV-2 .... S9
SARS-CoV-2, the *Coronaviridae* family and quadruplexes S13
SARS-CoV-2, the Virus Realm and quadruplexes S17
Biophysical Experiments S20
4. Other bioinformatic figures S23
5. Data results S25
G4-iM Grinder SARS-CoV-2 annotation files S25
G4-iM Grinder Analytical data S25
G4-iM Grinder RAW data S26
6. References S27

1. G4-iM Grinder, methodological scheme and upgrades

G4-iM Grinder algorithm

G4-iM Grinder is an algorithm designed to find and evaluate potential Quadruplex sequences in DNA or RNA. As with other modern quadruplex algorithms, it is composed of two parts: a search engine (that grants sensitivity) and an evaluation process (that grants specificity).

A. **Search engine:** G4-iM Grinder's search engine is very adaptable and can accept different variables (a quadruplex definition set) to detect all the arrangements that can give rise to a quadruplex. Although the algorithm is based on the Folding Rule $\{GGG(X_{1-7}GGG)_3\}$[1,2], it has been adapted to detect potential quadruplexes with bulges in their G-runs, longer loops, smaller G-runs, alternating G-runs, higher-order quadruplex versions and other features [3]. G4s with these characteristics have been studied and confirmed to form *in vitro*, yet few algorithms can to detect them.

G4 iM Grinder's search engine is capable of finding all possible sequences by using the appropriate quadruplex definition set. The search engine was intended for this purpose, to find everything that fits the quadruplex definition.

These are all the genome's Potential Quadruplex Sequences (PQSs or PiMS).

Considerations:

The results obtained are dependent on the quadruplex definition set (configuration) introduced to the search engine. As sensitivity, not specificity, is its sole function, ideally a very lax configuration of parameters should be introduced to it as predefined. For example, the *lax* configuration used here or even laxer configurations. However, this comes at the cost of requiring more computation power. For longer genomes with high G/C content the analysis' time, disk space and RAM requirements may be too demanding for personal computers.

B. Scoring systems: These algorithms are used to predict the probability of the results found by the search engine to form quadruplex structures. They grant specificity to G4-iM Grinder. The default of the package, and what we used in the manuscript, is the average of PQSfinder's and G4Hunter's scoring algorithm results (variable *Score*). Each of these evaluate different parameters of the PQS [4,5]. PQSfinder considers the number of tetrads, the bulges in the runs and the length of loops of the sequence, whilst G4Hunter evaluates its G richness and C skewness.

To adapt the scoring system of G4Hunter (from -4 to 4) to PQSfinder, we multiplied G4Hunter's scale by 25 and implemented it in G4-iMGrinder. The scale ranges from -100 (C-rich sequences; very probable to form i-Motifs), to 100 (G-rich sequences very probable to form G4s), with an intermediate point at zero for sequences with low or NULL probability of forming quadruplexes. Hence, the higher the absolute value of the score, the more likely these can compete against other regular secondary structures for formation and give rise to a G4 (if its positive) or an i-Motif (if its negative).

Considerations:

The score systems output is a value that estimates the probability of the results found by the search engine to form quadruplex structures. Other algorithms classify the results in a binary manner with a minimum threshold, to return to the user only those that are to be considered G4s. PQSfinder includes a variable "*min_score*", to apply such threshold. G4Hunter, and other algorithms based on the sliding window, are conceptually based in this idea. On the contrary, QGRS mapper and other algorithms do not apply any threshold, and instead leave the decision to the user. This makes the threshold optimization non-critical to find candidates. G4-iM Grinder is included in the latter group.

As the score represents a probability, the scale of G4-iM Grinder contemplates more than the binary classification into forming or not forming a G4 or i-Motif. Instead, it classifies them into a high, medium and low probability of forming quadruplexes ($|\text{scores}| \geq 40$, ≥ 20 and < 20 respectively).

The threshold of 40 was selected as the higher margin because it displayed the best compromise between high precision and false discovery rates in the analysis of the G4-related sequences in the GiG database (version 2.0). Hence, it was established that any PQS with this score or higher can (probably) form G4s. In G4Hunter's scale terms, this threshold is 1.6 $\left(\frac{40}{25}\right)$.

However, false positives and false negatives still do happen. For example, 93 G4s included in the database of G4-iM Grinder have a lower score than the threshold of 40, and hence are false negatives when evaluated by this threshold. Of these G4s, all except one scored in the range of 20 to 40. Therefore, another threshold was established at a score of 20. The candidates that score between 20 and 40 have a lower chance of forming than the higher tier PQSs, but still have a probability of forming. In this range, higher false positives are also expected to be encountered. In G4Hunter's scale terms, this threshold is 0.8 $\left(\frac{20}{25}\right)$.

C. Other factors for evaluation: Besides the score, other factors can influence the study of a potential quadruplex. Some of the characteristics currently implemented in G4-iM Grinder are:

- Study of the frequency of appearance of the sequences, as to identify where and how many times a candidate is repeated in a genome.
- Presence of sequences that are already confirmed to form G4 and i-Motifs *in vitro* within its results.
- Presence of desired patterns in the candidates' loop/s.
- Biological landmark co-localization.
- Localization and analysis of potential higher-order quadruplex sequences.

D. *in vitro* evaluation: G4-iM Grinder (and all other quadruplex algorithms) makes predictions that need to be confirmed by *in vitro* experimentation (reviewed by [6]). Results that have known G4s or i-Motifs in their sequences have been successfully evaluated already elsewhere in the literature.

Methodological scheme

Fig. 1. Proposed workflow scheme with G4-iM Grinder

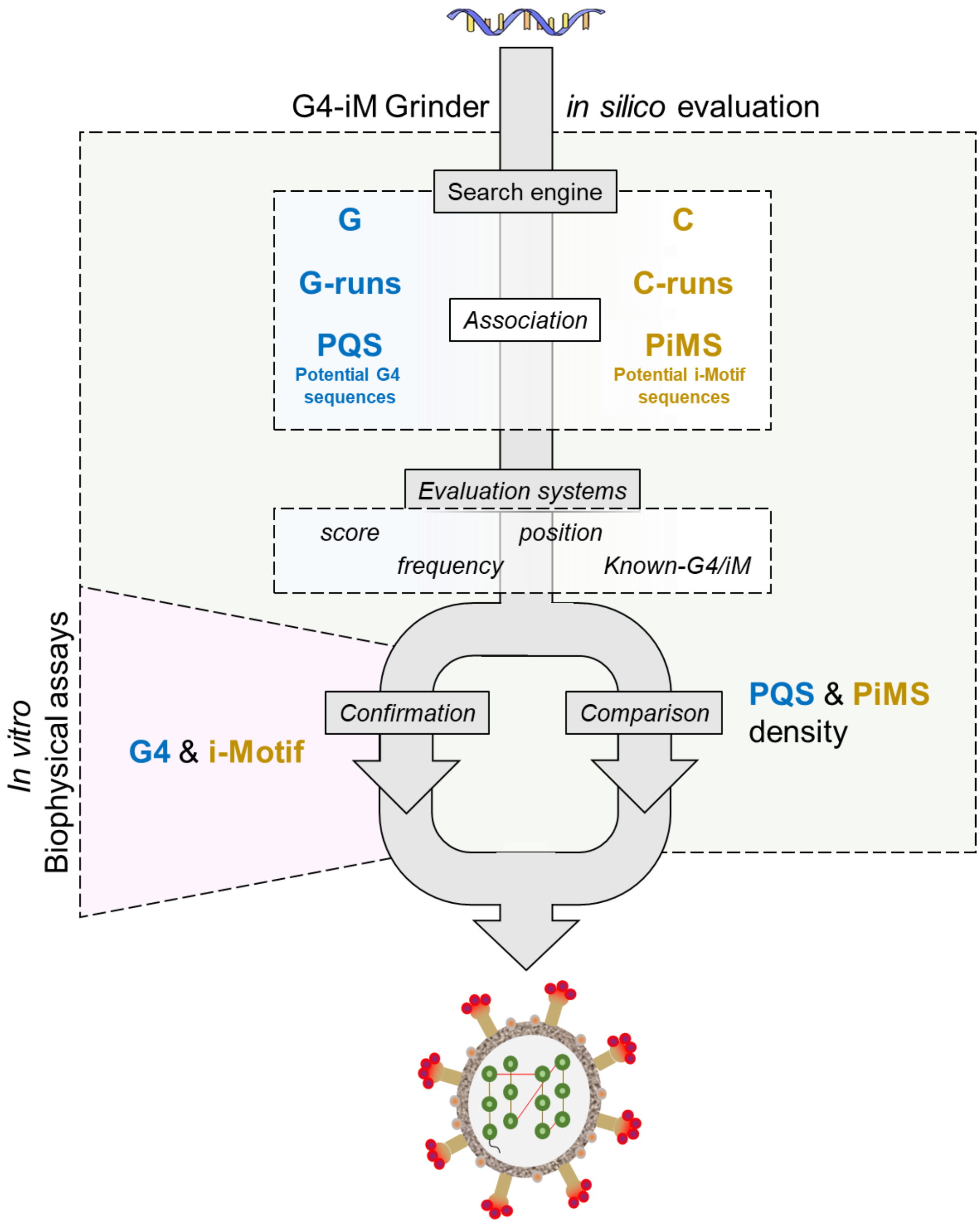

<u>Upgrades</u>

In order to extract more information and better analyse the viruses, G4-iM Grinder was updated. Two new functions were developed and are now incorporated in the GiG-package (as of GiG version 1.6.0) named *GiG.Seq.Analysis* and *GiG.df.GenomicFeatures.*

A. *GiG.Seq.Analysis* function is useful for retrieving the genomic characteristics of the sequence to analyse with GiG. This function can be used before a GiG analysis to determine the best search parameters to obtain quadruplex-related results. The function's outcome is a data frame with the most relevant genomic features, including length (in nucleotides), type of genome (DNA or RNA), strands (single or double), and G, C, T/U, A and N composition (as % of total sequence). The function also calculates the total number of runs with different conditions (predefined parameters, bulges per run: zero and one-quantities; run lengths: two to five and three to five-length) in the genome, and returns it to the user as total counts or genomic density.

B. *GiG.df.GenomicFeatures* function is suitable for determining the genomic features that share their location with (and hence may be affected by) GiG's PQS and PiMS results. It requires of an annotation file for the sequence, with which then will match positions. The function returns a data frame of all the matches found for the input sequences with all the information of the genomic feature hit.

To help us locate any G4 or iM already studied in the literature, we updated the GiG's DataBase (*GiG.DB*) to version 2.5.0. The library now includes 2941 quadruplex-related sequences that can be identified within any of GiG's results. The database is categorized by the capability of the sequence to form or not form quadruplex structures (2207 do, 734 do not), their relation to G-based or C-based quadruplexes (2652 G4, 289 iM) and the genome type (1914 DNA, 1027 RNA). The reference information (including DOI and/or PubMedID) of each sequence is also listed and accessible to facilitate further studies.

2. Genomes used

The reference genome of the SARS-CoV-2 (assembly ascension was GCF_009858895.2 released 13 January 2020) was downloaded from the NCBI database (https://www.ncbi.nlm.nih.gov/). All other genomes used, except those otherwise stated, were downloaded from the NCBI database via the biomartr R package. Further information regarding these genomes can be found within the data results section (Section 5, Data results).

The 17312 different SARS-CoV-2 viral genomes sequenced during the pandemic (from December-2019 to January-2021, by different laboratories worldwide) were retrieved from the online database GISAID [8]. These genomes are the result of filtering the database by their coverage (<1 % N content), completeness (>29000 nucleotides) and association to a clinical patient history (only those that have it). Further information regarding these genomes can be found within the data results section.

3. Results

SARS-CoV-2

G4-iM Grinder's analysis of the SARS-CoV-2 reference genome revealed 323 PQS and 189 PiMS candidates. As none |scored| over 40 (high probability of formation), we focused on the candidates with |scores| higher than 20 (medium probability). This filter resulted in the detection of 71 PQSs, 7 of which also scored over 30 (22 and 2 %, respectively). Regarding PiMS, 35 scored -20 or less, and 10 below -30 (19 and 5 %, respectively). In both cases, all the candidates were unique (only occurring once in the SARS-CoV-2 genome) and none included confirmed G4s or iMs (listed in the *GiG.DB* V.2.0) within their sequence.

We studied the potential biological effect of these candidates by determining their location and distribution within the genome of the SARS-CoV-2 (main text, Fig. 2). All PQSs with scores over 30, except one (found in the N gene), were located in the orf1ab polyprotein gene (in the nsp 1, 3 and 10 areas). Similarly, all the high-scoring PiMS were located in the orf1ab gene (in the nsp3, 4, 12 and 13 areas), except a candidate located in the 5' UTR region. When we lowered the |score| filter to 20, most results were identified as part of the orf1ab polyprotein. However, some PQSs were also found as part of the S, orf3a, M, orf8 and N genes and some PiMS as part of the S, orf3a, N genes and 3' UTR. When no score-filter was applied, nearly all biological features presented quadruplex candidates, with the exception of orf7a, orf7b and orf10, as well as the orf6 gene in the case of PIMS.

For each PQS and PiMS detected on the reference SARS-CoV-2, we studied its conservation rates using different SARS-CoV-2 genomes sequenced by different research groups/laboratories/institutions at different times and locations of the pandemic. In total, we analysed 17312 different SARS-CoV-2 genomes associated with clinical cases of CoVID with morbid or mortal outcomes. On average, the PQS conservation rate for all candidates was 97.7 ± 10.8 %, whilst for PiMS it was 94.5 ± 17.6 %. For results that scored at

least 20, PQS conservation decreased to 96.8 ± 13.2 % and PiMS increased to 96.9 ± 14.1 %. The mean sequence identity between the genomes was of 99.83 %.

The relationship between the candidate's conservation rate and the biological features potentially affected by the PQS and PiMS were then explored. On the one hand, we found that the less conserved sequences are located mainly in the 5' UTR section, where conservation can fall to 9 %. On the other hand, the 3' UTR region showed higher conservation levels with minima of 90 %. The rest of the sequences found in most of the other landmarks presented very high conservation rates, ranging between 99 and 100 % (285 PQS out of 323, 133 PiMS out of 189). Some exceptions exist, however. For example, a PiMS was found in the orf1ab gene with a conservation rate of 10 % (position 14391, nsp12 within the coronavirus RPol N-terminus) and three PQSs were located in the N gene with conservation rates of 34 %.

In a wider context, the total number of PQSs found in the 17312 different SARS-CoV-2 genomes ranged from 291 to 333 sequences (reference genome is 323). The PQSs that scored at least 20 ranged from 61 to 80 sequences (reference genome is 71). Although none of them presented already confirmed G4 sequences, eight PQSs with a high probability of forming G4 were found in SARS-CoV-2 genomes of Clades S, Lineage A.3 (main text, entry 7 Fig. 3A).

The total number of results for PiMS were in the range of 168 to 200 sequences (reference genome is 189). The PiMSs that scored -20 or less ranged from 27 to 40 sequences (reference genome is 32). 67 PiMSs with a high probability of forming i-Motifs were identified in SARS-CoV-2s of Clades GR, Lineage B.1.1.x (main text, entry 9 Fig. 3A). Several SARS-CoV-2 genomes (Hangzhou/ZJU-01-2020, Hangzhou/ZJU-03-2020 and Hangzhou/ZJU-08-2020) presented long repetitions of C-tracks in the start or end regions. In its DNA version, these C-tracks can form the C15, C18, C21 [9] and ATXN2L [10] iMs when protonated. The RNA version of the iM can also form, although with a lower stability [11].

158121 new PQS and PiMS were found in the 17312 SARS-CoV-2 that were not found in the reference genome. These variants were mainly located in the 5'UTR area and scored poorly (**Fig. 2**, A).

However, 5429 PQSs and 3298 PiMS variants were identified that |scored| at least 20. These are scattered in different areas of the genome and especially in the N and 3'UTR regions (**Fig. 2**, B).

The number of candidates and their differences from the reference genome were studied further in regards to their clade and lineage.

The GR clade showed a significant decrease in the number of PQSs and PQSs that |scored| at least 20 compared to the other clades. In addition, variant PQSs were also significantly less abundant here than in the other clades analysed. The GR clade had 0.16 PQS variants per genome analysed, whilst the S, GV, L and GH presented 1.44, 0.70, 0.70 and 0.58, respectively. The PQSs that |scored| at least 20 within the B.1.1.x lineage (where x is all the subgroups inside) of clade O and clade G were also significantly smaller than their general clades' results. On the contrary, the S clade in average had one more PQS that |scored| at least 20 than the reference genome (71 PQSs), and two more than the average of the analysis. The S clade's high number of variants per genome analysed together with the higher number of PQSs found, makes the clade divergent from the reference genome and the rest of the clades analysed.

For PiMS, the differences between clades and lineages were smaller and more constant between groups (**Fig. 2**, C). The G and S clades presented the highest numbers of variants per genome analysed (0.29 and 0.28 respectively) whilst clade GR presented the smallest (0.12). On average, 34 to 35 PiMS with a |score| of at least 20 were found for all clades and Lineages (35 PiMS in the reference genome), except for lineage B.1.258.x (where an additional candidate was identified) and lineage B.1.416.x of the same clade (where two less were found).

Fig. 2, A. PQS and PiMS variant counts per location in the SARS-CoV-2 genome (30 bins). **B**. PQS and PiMS variant counts that |scored| at least ≥ 20 per location in the SARS-CoV-2 genome. **C**. Centre, SARS-CoV-2 phylogenetic tree (by clade and lineage) of the sequences analysed. Lineages with less than 100 genomes were grouped (suffix x). Inner segment, Lineage: PiMS count (*A*), PiMS with |score| ≥ 20 (*B*) and PiMS divergence from the reference genome (*C*). Centre segment, Mean lineage percentage sequence identity with the reference genome (dots) compared to the overall mean found for the 17312 sequences analysed (black line). Outer segment, Clade: PiMS count (*A*), PiMS with |score| ≥ 20 (*B*) and PiMS divergence from the reference genome (*C*).

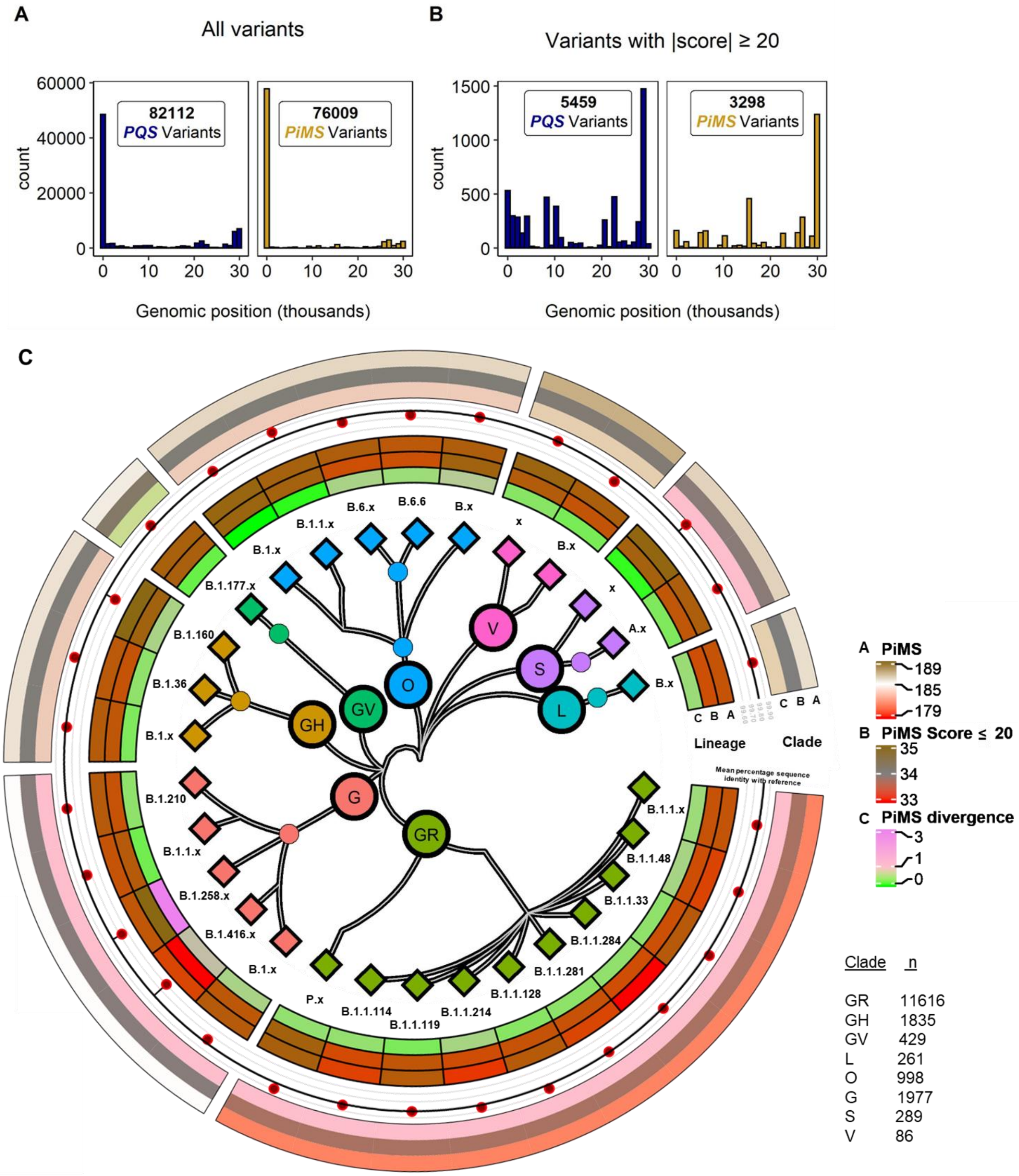

SARS-CoV-2, the *Coronaviridae* family and quadruplexes

We then explored the relationship of the SARS-CoV-2 with the rest of *Coronaviridae* family members. The most similar genomes found to that of the SARS-CoV-2 were the SARS- coronavirus (SARS-CoV) and the Bat coronavirus BM48-31/BGR/2008 (Bat-CoV-BM), with pair-alignment score values of 79 and 75 % respectively. These two genomes also presented several PQSs and PiMSs in common with SARS-CoV-2. The common candidates were analysed and their potential biological influence within the SARS-CoV-2 genome determined.
For PQSs, nine sequences are common between all three genomes (SARS-CoV-2, Bat-CoV-BM and the SARS-CoV), and can be found in the 5' UTR, 3' UTR and in the middle of the polyprotein orf1ab regions of the SARS-CoV-2. Eleven other sequences are present only amongst the Bat-CoV-BM and are located within the 5' UTR region, near to the orf1ab starting positions or in the middle of the orf1ab polyprotein. Eight additional PQSs are present only amongst the SARS-CoV and appear in the orf1ab gene and 3' UTR region of the SARS-CoV-2.

For PiMS, only one sequence common to all three genomes is located in the middle of the orf1ab gene. Five other PiMSs are present only amongst the Bat-CoV-BM are found in the 5' UTR region, near to the orf1ab starting positions or in the middle of the orf1ab polyprotein. Nine additional sequences are common only with the SARS-CoV and can be found in the start (less than 40 nucleotides away from the UTR; 2/9) and in middle of the polyprotein orf1ab gene (4/9), the E gene (1/9), and the N gene (2/9) of the reference SARS-CoV-2.

The common PQS and PiMS's position difference between the SARS-CoV-2 and the SARS-CoV is 59 ± 47 nucleotides, whilst the difference between the SARS-CoV-2 and the Bat-CoV-BM is 133 ± 109 nucleotides. The scores of these common sequences are however rather poor. None of the PiMS scored less than -20 and only four PQSs scored more than 20 (at least medium probability of formation; **Fig. 3**, A). Of these, the best candidate scored over 30, (entry 2 in **Fig. 3**). In general, the sequences presented small run

sizes, a high number of bulges, long loop lengths and a high number of complementary nucleotides within, which lowered their probability for quadruplex-formation.

We focused on the highest scoring sequences found in SARS-CoV-2 but not common to the other SARS-CoV and Bat-CoV-BM, and identified the variations in their sequences (**Fig. 3**, B). To do so, the previously found common PQS and PiMS were used as fixed points of a genomic-alignment to retrieve the tracks of interest that contain the potential quadruplex sequences.

For PQS1, the changes between the three species occurred in four different nucleotides, three of which are loops. In the Bat-CoV-BM version, the fourth G-run is annulled by the substitution of a G for a C, although a different G-run exists that can still allow the potential quadruplex formation. This PQS, however, has more bulges, longer loops and an increased number of C, which will make it less stable if formed. The SARS-CoV-2 and SARS-CoV versions have a potential fifth G-run domain downstream which can potentially interact with the PQS.

For PQS2, modifications are greater between species and occur in the central loop and second run area. In SARS-CoV, these modifications allow for a more potentially stable PQS derived from the incorporation of an extra 2-residue G-run. The Bat-CoV-BM version of the PQS has the majority of the central loop deleted and second run invalidated. These modifications, however, do not impede the potential formation of the quadruplex since a nearby G-run downstream can give rise to an alternative PQS.

For PiMS 3, the modifications directly prevent the potential formation of the iM in both SARS-CoV and Bat-CoV-BM genomes. PiMS 4 showed a greater level of conservation. Only two modifications between species occur and are located in the first and third loops. The Bat-CoV-BM version includes an extra C in the last C-run, which makes the PiMS more potentially stable than in the other family versions.

Fig. 3. A. PQS sequences found with a score of over 20 and common to the SARS-CoV-2, SARS-CoV and/or Bat-CoV BM48-31/BGR/2008 (entry 1 to 4), or which are common to other non-related viruses (entry 5 and 6). G-runs are in blue, C-runs are in yellow, loops are in red and bulges within the runs are in green. For each entry, the biological feature column lists the genomic landmark that hosts the potential quadruplex in the SARS-CoV-2. The percentage of conservation of each entry (between 17312 different SARS-CoV-2 genomes sequenced at different places and times during the 2019-2021 epidemic) is also given. **B**. Alignment of four PQS and PiMS with the highest |scores| found in the SARS-CoV-2 but not in the SARS or Bat coronavirus. Other common PQS and PiMS were used as fixed points to align the intermediate genomes, with which then to find the locations and variations of these potential structures within all three genomes. Nucleotides in blue and yellow are the potential G- and C-runs respectively that may give rise to the G4 or iMs. The nucleotides that are different from the reference SARS-CoV-2 genome are depicted in red. G or C nucleotides that may contribute to a nearby sequence are depicted in green.

A.

Entry	Start	Sequence	Score	Biological feature	SARS-CoV-2 conservation (%)	Other genomes
1	246	GGGUGUGACCGAAAGGUAAGAUGG	21	5'UTR	99.62	SARS-CoV & Bat-CoV
2	13385	GGUAUGUGGAAAGGUUAUGG	31	orf1ab - nsp10	99.92	Bat-CoV
3	13385	GGUAUGUGGAAAGGUUAUGGCUGUAGUUGUG	20	orf1ab - nsp10	99.89	Bat-CoV
4	15438	GGUCAUGUGUGGCGG	24	orf1ab - nsp12	99.81	SARS-CoV
5	10015	CCAACCACCACAAACC	-36	orf1ab - nsp4	99.77	Murid betaherpesvirus 1<br>Macropodid alphaherpesvirus 1<br>Vibrio phage douglas 12A4
6	28903	GGCUGGCAAUGGCGG	34	N	99.08	Mycobacterium phage Omnicron

B.

PQS 1: *Figure 2, entry 4*

**SARS-CoV-2**	Score: 34	start: 3451	GUUUACCUUAAACAU	GGAGGAGGUGUUGCAGG	AGCCUUAAAUAAGGC
**SARS-CoV**	Score: 34	start: 3384	AUACACCUGAAACAU	GGUGGUGGUGUAGCAGG	UGCACUCAACAAGGC
**Bat-CoV**	Score: 22	start: 3259	AUACACUUGAAACAU	GGUGGUGGUGUUGCACG	AGCACUAGAUAAAGC

PQS 2: *Figure 2, entry 7*

**SARS-CoV-2**	Score: 34	start: 28888	UUCUCCUGCUAGAAU	GGCUGGCAAUGGCGG	UGAUGCUGCUCUUGC
**SARS-CoV**	Score: 36	start: 28737	UUCUCCUGCUCGAAU	GGCUAGCGGAGGUGG	UGAAACUGCCCUCGC
**Bat-CoV**	*Not PQS*	start: 28276	AUCACCUGCACGCAU	GGCUGCC---GGAGG	AGAUACGGCACUUGC

PiMS 3: *Figure 2, entry 9*

**SARS-CoV-2**	Score:-36	start: 4875	CACU----AGUAA-U	CCUACCACAUUCCACC	UAGAUGGUGAAGUUA
**SARS-CoV**	*Not PiMS*	start: 4802	CACUCUGGAGAGC-C	CCGUCGAGUUUC-AUC	UUGACGGUGAGGUUC
**Bat-CoV**	*Not PiMS*	start: 4700	CAGU----AGGAAAU	GUUAUAGAAUUUCACA	UGGAAGGUGAAGUUC

PiMS 4: *Figure 2, entry 14*

**SARS-CoV-2**	Score:-38	start: 15909	UGAUGAUUAUGUGUA	CCUUCCUUACCCAGAUCC	AUCAAGAAUCCUAGG
**SARS-CoV**	Score:-37	start: 15839	AGAUGAUUACGUGUA	CCUGCCUUACCCAGAUCC	AUCAAGAAUAUUAGG
**Bat-CoV**	Score:-42	start: 15738	AGAUGAUUACGUGUA	CCUGCCUUACCCAGACCC	AUCUAGAAUUUUAGG

We analysed the candidates presence and relevance in the family of genomes through a more macroscopic perspective (**Fig. 4**, A). PQS density between *Coronaviridae* species lied between 705 to 3381 PQSs per 100000 nucleotides, with a mean of 1802 (PQS density for SARS-CoV-2 is 1080). Rousettus bat coronavirus, Rat coronavirus, Parker and Beluga whale coronavirus SW1 had the highest densities in the classification, whilst the human coronavirus HKU1 and several other HKU-CoV had the lowest. All these results were unique and therefore are repeated only once in the genome. 62 % (28 out of 45) of the family presented one or more PQSs with a score of over 40 (most probable PQSs to form quadruplex). The 2G_L1.NAR [12] G4 (in its RNA variant) was found within nine PQSs in the Wigeon-CoV HKU20 genome.

For PiMS, the range for the density of *Coronaviridae* family was in between 142 and 4938 sequences per 100000 nucleotides, with a mean of 1030 ± 929 (SARS-CoV-2 PQS density is 632). Magpie-robin coronavirus HKU18, Sparrow coronavirus HKU17 and Porcine coronavirus HKU15 topped the classification with densities of 4938, 3933 and 2930 respectively. Wencheng Sm shrew coronavirus was the least dense genome, followed by the Human coronavirus HKU1 and Human coronavirus OC43. 64 % of all members (29 out of 45) presented within their results at least one PiMS with a score of -40 or less (most probable PiMS to form *in vitro*). None had already confirmed iMs within their sequence and all were unique, being repeated only once in the genome.

SARS-CoV-2, the Virus Realm and quadruplexes

We further expanded the search to the remaining viruses classified in the NCBI database and found several PQS and PiMS in common with the reference SARS-CoV-2. These had a good score and high *inter*-SARS-CoV-2 conservation rates (**Fig. 3**, A entry 5 and 6). All of these matches were made with Group I viruses (of the Baltimore classification; dsDNA viruses). One PQS candidate located in the N-gene and CDS region of the SARS-CoV-2 was found in common with a Mycobacteria phage virus. This virus is known to infect the bacterial *Mycobacterium* genus that causes several diseases in humans (tuberculosis and leprosy). The PQS was identified as part of a scaffolding-protein gene within the Mycobacteria phage. Also, a PiMS located in the SARS-CoV-2's orf1ab gene is common to three different viruses; two from the *Herpesviridae* family that infects mammals (marsupials and murids) and a *Podoviridae* virus that infects bacteria from the *Vibrio/Aliivibrio* genus. The probability of these matches being due to randomness, taking into account the number of sequences analysed here, was calculated to be 5.00×10^{-5} and 1.25×10^{-13} for one and three matches respectively, and were assumed neglectable.

In Group IV (which includes the *Coronaviridae* family and 1309 other viruses), we explored the quadruplex presence and distribution per family (**Fig. 4**, B) and calculated their mean and standard deviation. These were then compared to the *Coronaviridae* results.

For PQS, the *Coronaviridae* family is in the lower range of the group's distribution. As a whole, the group's density was double of that found in the *Coronaviridae* family (means of 4034 and 1802, respectively). To the contrary, the *Flaviviridae* and *Tombusviridae* families showed the highest densities, with genomes that surpassed that of the *Coronaviridae* several fold. In particular for *Flaviviridae*, its mean PQS density was found to be more than four times that of *Coronaviridae* (means of 9079 and 1802, respectively). 106 viruses within the Group also presented already confirmed G4 sequences within their results. These were found in 36 species belonging to the *Flaviviridae* family, 9 to the *Picorbiridae*, 8 to the *Tombusviridae* and 7 to the *Closteroviridae* families, amongst others.

For PiMS, the *Coronaviridae* family was again in the low end of the group's distribution. The group's mean

density was over three times that of *Coronaviridae*'s (mean of 3731 and 1043, respectively). Contrastingly, the *Tymoviridae* family was found to be very rich in potential candidates (mean of 25359). Its results surpassed by over an order of magnitude most of the other group member results. The *Picornaviridae*, *Secoviridae* and *Luteoviridae* families also had very dense genomes, and within these four families, 9 virus species presented confirmed iMs in their genome (of 11 in total).

In a wider context, we investigated the prevalence and distribution of potential quadruplexes in the entire virus realm and compared them to Group IV's results. We also examined the results on a global scale (**Fig. 4**, C).

For PQS, we detected relatively small differences between the groups. Group I (dsDNA) viruses displayed the highest mean density followed by Groups III, II and VI. The entire virus realm density was found within the limits of 40 to 27687 PQSs per 100000 nucleotides with a mean of 4441. The *Herpesviridae* and *Siphoviridae* families from Group I and *Inoviridae* family in Group II displayed the highest PQS densities amongst the 6680 viruses analysed. Additionally, several G4s confirmed in the literature were found in 1372 viruses within Group I, 133 within Group II, 74 within Group III, 106 within Group IV, 18 within Group V, 8 within Group VI and 14 within Group VII (**Fig. 7**). Group differences for PiMS were also relatively small, with the largest mean being Group VI followed by Groups I, III and IV. Here, the virus realm density was calculated in the range of 0 to 51771 with a mean of 4221. The *Tymoviridae* family and other Group IV families, mentioned previously, topped the rank, together with some species of the *Herpesviridae* family from Group I. iMs confirmed in the literature were found in 175 viruses within Group I, 5 within Group II, 11 within Group IV, 2 within Group V and 2 within Group VI.

Fig. 4. A. Result density (per 100000 nucleotides) of PiMS (left) and PQS (right) within the *coronaviridae* family. The results are ordered in descending order of unfiltered result density. In grey, all-result density per virus. In light blue (for PQS) and light yellow (for PiMS), the density of structures with at least medium probability of formation (|S.| ≥ 20). In intense blue (for PQS) and intense yellow (for PiMS), the density of structures with at least high probability of formation (|S.| ≥ 40). A base-10 logarithmic scale has been used for the x-axis to appreciate the big differences between results. **B**. Result density boxplots for all the families of viruses in the Baltimore classification group IV (+) ssRNA, which includes the *Coronaviridae* family. All families with less than ten members were merged into the '*Other Families*' group. A base-10 logarithmic scale has been used for the y-axis. Results are divided between PQS (left) and PiMS (right) and between score criteria (top- all result density, middle – result density with a medium probability of formation, bottom- result density with a high probability of formation. **C**. Result density boxplots for all the Baltimore classification groups. A base-10 logarithmic scaled has been used for the Y-axis. Results are divided between PQS (top) and PiMS (bottom) and between score criteria (left- all result density, middle- result density with a medium probability of formation, right- result density with a high probability of formation).

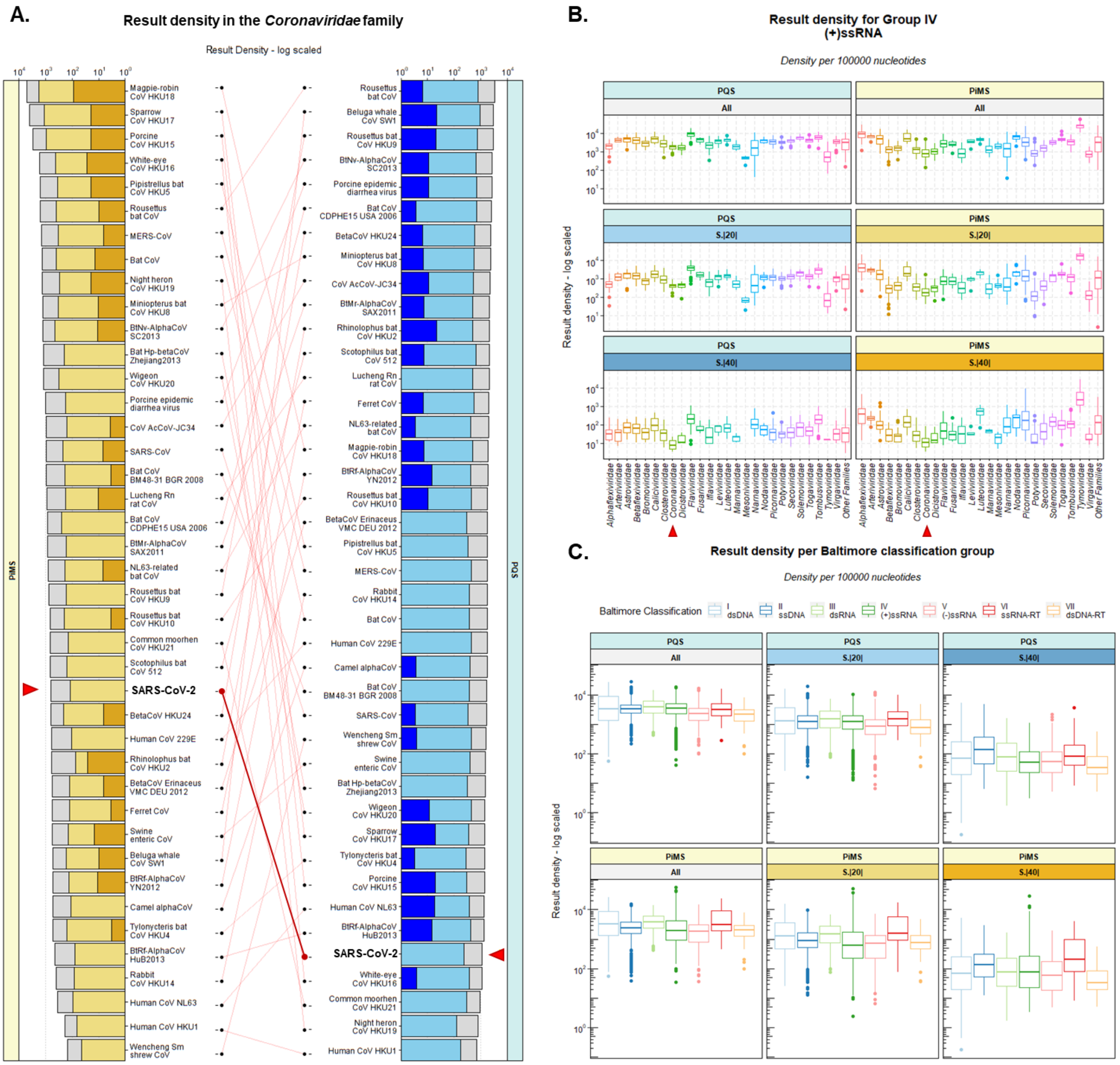

Biophysical Experiments

To validate the predictions of our bioinformatics search, we selected three candidates using the criteria mentioned in the material and methods section. Two of them were potential G4 forming sequences and the third one was an iM candidate.

The first G4 examined is found in the N-gene of the SARS-CoV-2 with a conservation rate of 99.08 % (CoVID-RNA.G4-1; main text, Fig. 4 A). The NMR spectra of this RNA exhibited a broad set of signals around 11 – 12 ppm, characteristic of guanine imino protons involved in G-tetrads. These signals are observed at high temperatures indicating that the G-quadruplex is quite stable (main text, Fig. 4, B). Additional signals around 12 – 13 ppm, which are characteristic of Watson-Crick base-pairs, can also be observed at low temperature. These interactions may arise from loops between G-tracts or alternative conformation such as hairpin-like structures. The CD spectra of the candidate revealed a positive band at 264 nm and negative band with a minimum at 240 nM consistent with the formation of a G4 of a parallel topology (main text, Fig. 4D, left). Melting experiments monitored by CD confirmed the great stability of the G4, whose melting temperature (Tm) was calculated to be 54.4 ºC at $[K^+]$ = 50 mM. Encouraged by these results, we additionally selected another candidate for experimental analysis with a very high conservation rate (CoVID-RNA.G4-2; main text, Fig. 4). This candidate is located in the orf1ab gene within the nsp3 region. As for the previous analysis, NMR and CD spectra revealed a stable parallel G4-quadruplex, with a CD-monitored Tm of 48.1 ºC. In this case, the CD spectra presented an additional band at 310 nm, most likely related to the association between two quadruplexes to form a dimeric structure. This is consistent with the number of imino signals observed in the NMR spectra at high temperatures, which suggests the presence of more than two G-tetrads.

In the case of PiMS, we selected a very conserved candidate (99.54 %) found in the orf1ab – nsp12 region of the virus (CoVID-RNA.iM-1; main text, Fig. 4 A). In NMR, only two small signals appeared in the 12.5 – 14.5 ppm range in the RNA version of the PiMS at neutral pH. Under acidic conditions more signals were observable, including a peak at 15 ppm which could be associated with ***C·C+*** iminos (**Fig. 5**, B). However, further analysis by 2D NMR spectroscopy revealed that this signal arises from an AU base pair (**Fig. 5**, D).

We must conclude that this sequence, although folded, does not form an iM. This result is not totally unexpected, since the lower stability of RNA vs DNA iM is well known. In spite of this negative result, and to check the capability of our algorithm to detect iMs, we decided to study the DNA version of this sequence (CoVID-DNA.iM-1; main text, Fig. 4 A). Most interestingly, the NMR spectra of this DNA oligonucleotide exhibited several imino signals in the 15 – 16 ppm range, characteristic of ***C·C+*** base pairs. These signals are observable in the 5.5 to 6.7 pH. Additionally, amino groups from ***C·C+*** (in the 9 – 10 ppm range) and TT base pairs (in the 11 ppm region) are also observable. As TT base pairs are common capping groups in iMs, it is interesting that the SARS version of this sequence (SARS-DNA.iM-1; **Fig. 5**, C), which only differs from SARS-CoV-2 in a single nucleotide within the first loop (from TT to TG), was unable to form an iM under all pH conditions studied (even at pH 5.4).

Fig. 5, 1H NMR spectra of CoVID-DNA.iM-1 (**A**) and CoVID-RNA.iM-1 (**B**). **C**, 1H NMR spectra of SARS-CoV version of CoVID-DNA.iM-1. **D**. Regions of NOESY spectra of CoVID-DNA.iM-1 (left) and CoVID-RNA.iM-1 (right). Characteristic cross-peaks between imino (15-16 ppm) and amino (9.0-9.5 ppm) protons of ***C:C+*** base pairs are indicated on the left panels. The cross-peaks observed in the RNA are very weak and, most probably, correspond to AU and GC Watson-Crick base pairs. Spectra were recorded with 150 ms mixing time (25 mM sodium phosphate, pH 5.1, T = 5ºC).

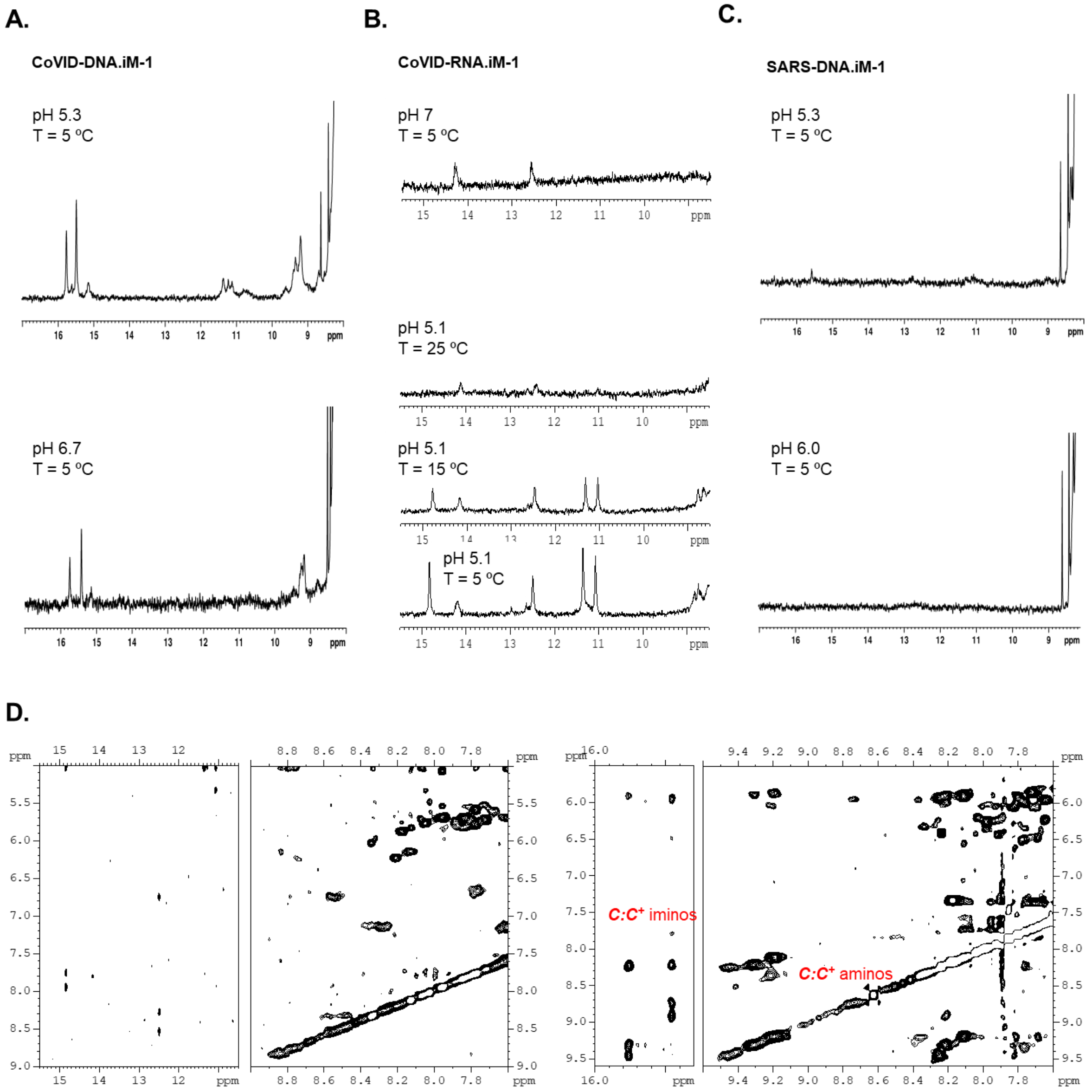

4. Other bioinformatic figures

Fig. 6, A. Run counts versus genome length of the 6680 viral genomes analyzed. **B.** Run density versus G|C % genomic content of the 6680 genomes analyzed.

Graphs are divided by quadruplex type (PQS and PiMS). For B, the graphs are also divided into perfect (in purple - left) and all runs (in green – right). Perfect runs include G and C-runs with no bulges, and with lengths comprehending between two and five nucleotides. All runs include perfect plus imperfect runs (with a bulge per run). For A, axes are log-scaled. Graphs include 2-dimensional density lair and best-fit linear model line. Correlation parameters and their significance are also given. Density per 100000 nucleotides.

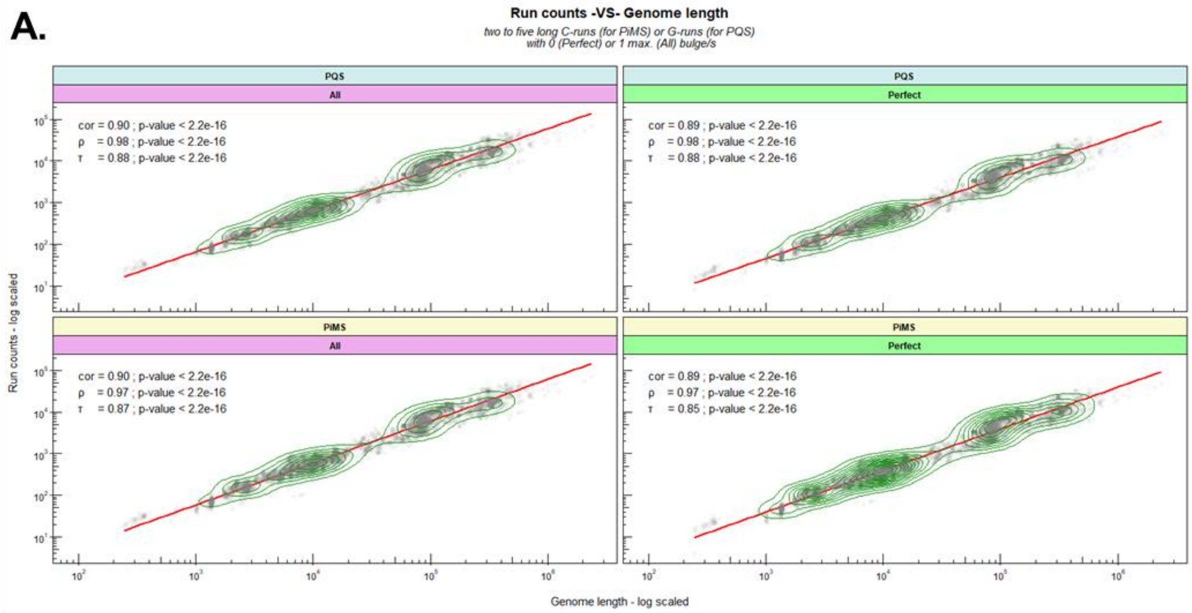

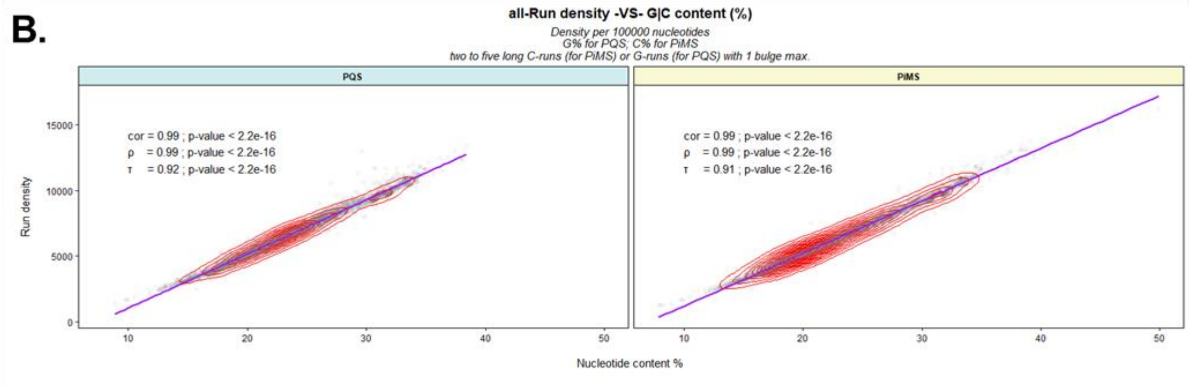

Fig. 7. Number of viruses with at least one confirmed G4 or i-Motif sequences in their DNA or RNA. The viruses are grouped per Baltimore classification groups and divided by quadruplex type (G4s and i-Motifs). Results are based on *GiG.DB* version 2.5.0.

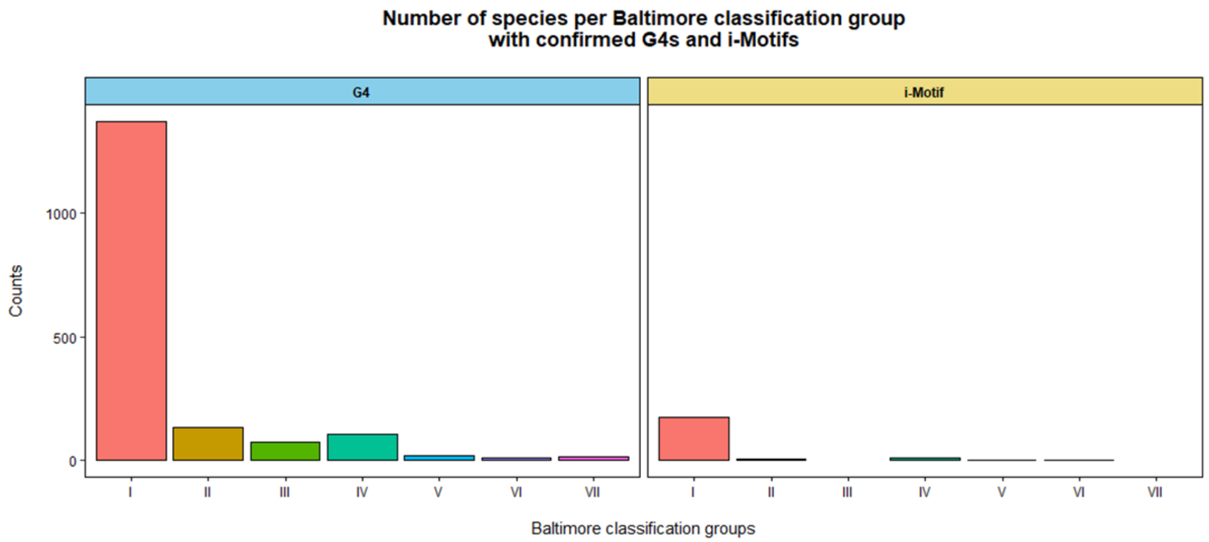

5. Data results

The data results for the entire analysis can be downloaded from G4-iM Grinder's GitHub webpage result section (https://github.com/EfresBR/G4iMGrinder). The package can be retrieved and installed also from here. The data is divided in three groups:

G4-iM Grinder SARS-CoV-2 annotation files

These include RDS (recommended), xlsx and gff3 annotation files of the analysis on the SARS-CoV-2 reference genome (GCF_009858895.2). It includes all the positions of the PQS and PiMS found, the conservation, and the G4s and iMs already confirmed in this work.

These include an RDS file of the PQS and PiMS found in other lineages and clades not found in the reference genome. Each entry has the GISAID ID, name, Lineage and Clade of the SARS-COV-2 genome where it was located.

G4-iM Grinder Analytical data

Analysis.RData, is the analysis results on the raw G4-iM Grinder data. It includes 3 lists.

1) *Analysis.Coronaviridae.fam* – Analysis with *GiGList.Analysis* function of the GiG-package of the *Coronaviridae* family. PQS and PiMS lists are the analysis for PQS and PiMS respectively. *df.index* data frame stores the identification of each genome used.
2) *Analysis.Virus.realm* - Analysis with *GiGList.Analysis* function of the GiG-package of the entire virus realm. PQS and PiMS lists are the analysis for PQS and PiMS respectively. *df.index* data frame stores the identification of each genome used. *Genome* data frame is the analysis with the function *GiG.Seq.Analysis*.
3) *Baltimore.C* – Baltimore Classification tables regarding each group characteristics and classification of each family into its group.

G4-iM Grinder RAW data

Virus.Results.RDS, includes the raw data of the G4-iM Grinder analysis on all the virus realm as a list. The list groups virus species by their families. Each species list includes a PQS and PiMS sublist. These store the composition, location, confirmed quadruplex-forming sequences presence and score (amongst others) of PQS/PiMS found in each virus. The information used in this analysis was Method 2; size restricted overlapping search method (PQSM2A data.frames), although Method 3 results are also included.

GISAID.REF.RAR, includes three PDFs with the references of the genomes used in the 17312 genomes analyzed of the SARS-CoV-2 as retrieved via the GISAID database.

6. References

1. Huppert JL. Prevalence of quadruplexes in the human genome. Nucleic Acids Research. 2005;33: 2908–2916. doi:10.1093/nar/gki609

2. Todd AK. Highly prevalent putative quadruplex sequence motifs in human DNA. Nucleic Acids Research. 2005;33: 2901–2907. doi:10.1093/nar/gki553

3. Belmonte-Reche E, Morales JC. G4-iM Grinder: when size and frequency matter. G-Quadruplex, i-Motif and higher order structure search and analysis tool. NAR Genomics and Bioinformatics, Volume 2, Issue 1, March 2020, lqz005, doi:10.1093/nargab/lqz005

4. Bedrat A, Lacroix L, Mergny J-L. Re-evaluation of G-quadruplex propensity with G4Hunter. Nucleic Acids Res. 2016;44: 1746–1759. doi:10.1093/nar/gkw006

5. Hon J, Martínek T, Zendulka J, Lexa M. pqsfinder: an exhaustive and imperfection-tolerant search tool for potential quadruplex-forming sequences in R. Bioinformatics. 2017;33: 3373–3379. doi:10.1093/bioinformatics/btx413

6. Kwok CK, Merrick CJ. G-Quadruplexes: Prediction, Characterization, and Biological Application. Trends in Biotechnology. 2017;35: 997–1013. doi:10.1016/j.tibtech.2017.06.012

7. Drost H-G, Paszkowski J. Biomartr: genomic data retrieval with R. Bioinformatics. 2017; btw821. doi:10.1093/bioinformatics/btw821

8. Shu Y, McCauley J. GISAID: Global initiative on sharing all influenza data – from vision to reality. Euro Surveill. 2017;22: 30494. doi:10.2807/1560-7917.ES.2017.22.13.30494

9. Školáková P, Renčiuk D, Palacký J, Krafčík D, Dvořáková Z, Kejnovská I, et al. Systematic investigation of sequence requirements for DNA i-motif formation. Nucleic Acids Research. 2019;47: 2177–2189. doi:10.1093/nar/gkz046

10. Wright EP, Huppert JL, Waller ZAE. Identification of multiple genomic DNA sequences which form i-motif structures at neutral pH. Nucleic Acids Research. 2017;45: 2951–2959. doi:10.1093/nar/gkx090

11. Snoussi K, Nonin-Lecomte S, Leroy J-L. The RNA i-motif. Journal of Molecular Biology. 2001;309: 139–153. doi:10.1006/jmbi.2001.4618

12. Abu-Ghazalah RM, Macgregor RB. Structural polymorphism of the four-repeat Oxytricha nova telomeric DNA sequences. Biophysical Chemistry. 2009;141: 180–185. doi:10.1016/j.bpc.2009.01.013
