# Supplementary material for: Potential G-quadruplexes and i-Motifs in the SARS-CoV-2
Source: PLoS One. 2021 Jun 8;16(6):e0250654. doi: 10.1371/journal.pone.0250654 (PMC8186786; doi:10.1371/journal.pone.0250654)
Supplement: Attachment — Submitted filename: DV.2.docx [file pone.0250654.s002.docx]

Dear Dr. Belmonte Reche,

Thank you for submitting your manuscript to PLOS ONE. After careful consideration, we feel that it has merit but does not fully meet PLOS ONE’s publication criteria as it currently stands. Therefore, we invite you to submit a revised version of the manuscript that addresses the points raised during the review process.

After careful review of the comments, it appears that the reviewers have a several issues with the manuscript. One of the main concerns was the use of the 'lax' setting for the G4-iM Grinder and the resulting predicition of G-quadreplexes based on this setting, as well as comparision with alternative G-quadraplex tools such as e.g. cGcC or G4Hunter.

Please address these concerns, along with the other comments made by the reviewers, in your re-submission.

Eric Charles Dykeman, Ph.D.
Academic Editor
PLOS ONE

Journal Requirements:

When submitting your revision, we need you to address these additional requirements.

Dear Reviewers and Editors,

Thank you for your comments and suggestions that will help us improve our manuscript. Before addressing the concerns of the reviewers one by one, we want to include some clarification on the main issues compiled by the editor. This will also help us answer the reviewer’s comments. We have divided this part of the response into three section for a better overall understanding of our manuscript:

**GENERAL REMARCKS:**

**G4-iM Grinder’s search engine, and Score evaluation system to find and discriminate between PQS candidates.**

To understand why we used a LAX configuration, and why the selection of this configuration does not negatively affect the results found with G4-iM Grinder, it is first necessary to understand how G4-iM Grinder works. A complete description of the algorithm can be found here (1).

G4-iM Grinder is an algorithm designed to find and evaluate potential Quadruplex sequences in DNA or RNA. As with other modern quadruplex algorithms (including PQSfinder, G4Hunter and QGRS mapper) it is composed of two parts: a search engine and an evaluation process.

G4-iM Grinder’s search engine is very adaptable and can accept 13 different variables to detect all the arrangements that can give rise to a quadruplex. Although the algorithm is based on the Folding Rule Set {${GGG\left( X_{1-7}GGG \right)}_{3}$}(2), it has been adapted to detect potential quadruplexes with bulges in their G-runs, long loops, smaller G-runs, alternating G-runs, higher-order quadruplex versions, etc (1). G4s with these characteristics have been studied and confirmed to form in vitro, yet most algorithms, other than G4-iM Grinder are currently not able to detect these structures. For example, PQSfinder does not allow for the study of Potential i-Motifs, higher order structures, fluctuating G-run structures or sequences under the score threshold. Additionally to most of that, QGRS mapper does not also allow bulges in its analysis. G4Hunter cannot be configured altogether because it uses a floating window method of analysis, different from the others mentioned here. In contrast, G4 iM Grinder’s search engine is capable of finding all these sequences by using the appropriate quadruplex definition set. The search engine was intended for this purpose, as we wanted to detect everything that could possibly form a quadruplex. These are all the genome’s Potential Quadruplex Sequences (PQSs).

The second part of the algorithm is the evaluation process of each sequence. The default of the package, and what we used in the manuscript, is the average of PQSfinder’s and G4Hunter’s scoring algorithm results (variable Score) (1). Both PQSfinder and G4Hunter scoring systems have been used efficiently multiple times as quadruplex prediction methodologies in literature (124 cites of G4Hunter as seen in the journal web page), and their prediction capabilities have been published and outperform those predicted by QGRS mapper score system (3). PQSfinder and G4Hunter algorithms evaluate different parameters of the PQS to give a probability of it actually being able to form a G4 *in vitro*. PQSfinder considers the number of tetrads, the bulges in the runs and the length of loops of the sequence. G4Hunter evaluates the G richness and C skewness of the sequence, in the range of -4 to 4.
To adapt the scoring system of G4Hunter to PQSfinder and G4-iMGrinder, we multiplied G4Hunter’s values by 25. This extended its range from -100 (C-rich sequences; very probable to form i-Motifs), to 100 (G-rich sequences very probable to form G4s), with an intermediate point at zero for sequences with low or NULL probability of forming quadruplexes. This scale is also G4-iM Grinder’s scale, and is depicted in Figure 1 of the article. The range of -100 to 100 is more understandable than -4 to 4. More information on the adaptation of G4Hunter, cGcC and PQSfinder algorithms in G4-iM Grinder can be found in the original article, supplementary material (1).

The result of the evaluation process is a score for each PQS found by the search engine. The higher the absolute value of the score, the more likely it is to form a G4 (if its positive) or an i-Motif (if its negative). With these values, we can now filter the search engine results and discriminate between those that are probable to form G4 or i-Motifs in vitro, than those that aren’t.

**LAX configuration ruleset:**

As the editors and reviewers can understand, one of the advantages of G4-iM Grinder is its capability of finding Quadruplexes that no other search engine can find. To do so, a LAX configuration needs to be introduced to the search engine. This will increase the sequences located in the genome, including the sequences not normally detected by other algorithms. Then, all of these will be evaluated for their potential to form Quadruplex structures by the scoring systems. This is the only way to locate the sequences already found in other viruses such as VIH (with 2G-sized runs) (4) , EBOLA virus (with bulges in runs) (5), with longer loops or the ones found in this manuscript regarding the SARS-2-CoV. As commented by the reviewers, The LAX configuration will also increase the number of sequences found which most likely will not form G4s. These are discriminated efficiently by the evaluating scoring systems. When analyzed by their G4-probability of formation, defects (like smaller runs, bulges in between runs, longer loops and longer size of the sequence) are considered negatively in their evaluation (3, 6).

Ideally, the LAX configuration ruleset should be the predefined setup for all searches with G4-iM Grinder, as to take advantage of the full potential of its search engine. Then, the results will be evaluated by the scoring systems, and then filtered by their actual probability of forming Quadruplex structures. However in reality, the burden of the analysis and the resulting data can be too demanding for current personal computers (Answer 3 to Reviewer 2.) Currently only viruses can be analyzed in bulk under the LAX configuration ruleset with moderate and sensible time requirements because of their small-sized genomes.

**Scoring system and its classification, used to discriminate between the search engine results (PQSs) in terms of their probability to form quadruplexes *in vitro.***

With the final G4-iM Grinder results, where all the PQS have been found and evaluated, we then have to decide what sequences to focus on. Usually, the attention will be focused on those PQSs with high score values and therefore with the highest probability of forming G4s *in vitro*. As most algorithms designed to evaluate quadruplex sequences are not binary classifiers (including G4Hunter, PQSfinder, QGRS mapper), but rather return a value whose magnitude is directly proportional to the probability of forming G4s, a threshold is needed to do the binary classification. This threshold criterion differentiates PQS between “will form G4”, and “won’t form G4”.

In G4Hunter, the authors considered the threshold in the range of 1 to 2, to distinguish between sequences that are predicted to form G4 from those that not (in their scale of -4 to 4). These parameters are commonly used in literature nowadays, especially the 1.2 and 1.5. These values were obtained by calculating the best compromise between precision and false discovery rates in a set of over 200 sequences that are known to form (or not form) G4s *in vitro*. However, PQSfinder authors found that the best threshold criterion for G4Hunter was 0.7 (3). In terms of G4-iM Grinder’s scale (which is just G4Hunters scale multiplied by 25) the threshold range is of 25 to 50. The common thresholds of 1.2 and 1.5 are 30 and 37.5, respectively. PQSfinder authors suggested 17.5 for G4Hunter.

In the original article of G4-iM Grinder, we used G4Hunter’s range as our starting reference. The threshold of 40 was selected as the higher margin because it displayed the lowest False Positives events for the detection of G4s in G4Hunter. Hence, it was established that any PQS with this score or higher can (probably) form G4s.
As the threshold is very stringent, especially compared with the thresholds proposed by G4Hunter and PQSfinder, we also established a lower margin at a threshold of 20. Between a score of 20 and 40, the PQSs have a lower chance of forming than the higher tier PQSs but still have moderate chances of forming. 20 was selected because it’s the mid-way point of 40 and 0 (between high and infinitesimal probability to form G4s), which was also near the midpoint of the lower range margin proposed by G4Hunter and that proposed by PQSfinder authors regarding G4Hunter (25 and 17.5, respectively).

These scale thresholds were further studied with a larger database of quadruplex related sequences within G4-iM Grinder (which now contains over 2000 G-quadruplex, i-Motif, DNA and RNA related sequences).
For G-quadruplex and related sequences, these were evaluated by all the scoring systems and the criteria threshold was mapped to obtain the best compromise between high precision and false discovery rates (in terms of Matthews Correlation Coefficient (phi coefficient or MCC); Figure N1, A). G4-iM Grinder scores (which is the mean of G4Hunter and PQSFinder) displayed better than the individual use of the algorithms. These seem to complement each other’s prediction as seen by the overall results ratios (Figure N1, B). As both G4Hunter and PQSfinder consider different aspects of quadruplex formation in their evaluation process, the average of both improves the performance in the prediction classification. G4-Hunter’s threshold criteria were also lower than expected, as commented on by PQSfinder authors.

Figure N1, **A.** Results of mapping the best threshold criteria for the discrimination of PQS into forming G4s or not forming G4s, per scoring system. Matthews Correlation Coefficient (phi coefficient or MCC) was used to find the highest threshold criteria. The scoring systems include SCORE (the G4-iM Grinder score, which is the average of G4Hunter and the adapted algorithm of PQSfinder), G4Hunter, pqsfinder (PQSfinder algorithm adapted for G4-iM Grinder) and pqsfinder.ORI (original version of PQSfinder). **B.** Results of the highest MCC threshold criteria, their result ratios and confusion matrix for the analysis of 1490 G-quadruplex related sequences.

A

B

It can also be appreciated that although the parameters of the scoring algorithms are good, false positives and false negatives still do happen. For the score of G4-iM Grinder, these have been mapped mostly from the 40 to 20 score range (Figure N2). 93 G4 sequences confirmed *in vitro* (included in the database of G4-iM Grinder) have a lower score than the threshold of 40, yet only one G4 Sequence has a score of less than 20. The latter is the “GGCCATGGTCCTCCAGGTCCCCATGG” G-quadruplex named **bswR** [10.1128/mBio.02926-19].

Figure N2, Density plot of the G-quadruplex sequences included in G4-iM Grinder package by score (A), and by score for those that score between 40 and 20 (B). Plots are grouped by quadruplex type: In blue, sequences that form G4. In red, sequences that do not form G4.

**Comparison with other algorithms, such as G4Hunter and QGRS mapper.**

G4-iM Grinder can be compared to the other algorithms in regards to the search engine and the evaluation systems it uses.

The search engine results are dependent on the configuration we introduce to the algorithms.

The evaluating systems are dependent on the thresholds we filter them with.

In regards to the search engine (and hence the number of PQSs found in the genome), G4-iM Grinder is more adaptable than QGRS mapper and PQSfinder’s search engines (at least in their current versions) and it will always be able to find more PQSs (with the correct configuration). Furthermore, we can even mimic and reproduce their results with G4-iM Grinder.

For example, we could reproduce QGRS mapper predefined parameters which are:
Max PQS length:	30
Min G’s in G-runs:	2
Loop size range:	0-36	,with G4-iM Grinder’s search engine by using a configuration with these same values. We similarly could do this for PQSfinder. In either case, the LAX configuration is more flexible than all these other configurations, and hence it will always find more results if introduced to G4-iM Grinder. G4Hunter and G4RNA search engines are based on sliding windows instead of pattern matching, and hence cannot be reproduced directly by G4-iM Grinder’s search engine. The results here are filtered by the threshold directly, to be RAM efficient.

The SARS-CoV 2 genome is quite particular. Its genomic size and configuration, more than a poor G composition, is responsible for not finding folding rule abiding PQSs (that is that follow G_3-5_(X_1-7_G_3-5_)_3_). Most Gs are found as ‘two repetitions’ runs (2-G runs, GG) instead of the required three for the folding rule (3-G runs, GGG), when compared to other genome ratios.

Bartas and colleagues used G4 Hunter and QGRS mapper in the SARS-CoV-2 genome to localize PQSs (7). They found one PQS with G4Hunter using a threshold of 1.2. No PQSs were found with higher thresholds. Their results were confirmed with QGRS mapper with a configuration that allows 2-G runs. Here they found 25 PQSs. Ji et al. analysis was also based on QGRS mapper and hence found the same 25 PQS (8). As was (9). All of these results were found with G4-iM Grinder using the LAX configuration (except the PQS: “**GG**TTTTCCATTTAATAAAT**GGGG**TAA**GG**”, where QGRS mapper detects the GGGG sequence as two different runs, and GiG detects it as one), and over 40 more sequences that have equally moderate probabilities of forming G4. Many have actually been confirmed to form *in vitro* (including those in this manuscript).

Regarding the comparison between evaluating systems, G4-iM Grinder uses G4-Hunter and PQSfinders average as a scoring system. Both have been amply contrasted in literature as mentioned previously.

In the supplementary material, first section, the description made here was summarized regarding the search engine and scoring systems of G4-iM Grinder.

A new section was added in the main manuscript (PQS comparison) where we compare the different results obtained by other research groups using other quadruplex algorithms, with those found here.

**Reviewer #1:** Exploring G- and C-quadruplex structures as potential targets for the severe acute respiratory syndrome coronavirus 2
By Efres Belmonte-Reche* et al (*Corresponding author)
Submitted to PLoS One (Editorial No. PONE-D-20-34438)

General Comments
G- and C-quadruplex secondary structures (G4s and iMs, respectively) are found in the cellular genomes of many animal species and have complex roles in the regulation of metabolic pathways, including the biochemistry of telomeres and of oncogenic promoters (genome stability). Bacteria and many viruses have recently been explored for the presence of such structures in their genomes. Here, the genomes of SARS-CoV-2 isolates (of the reference strain, GCF_009858895.2, and of >3000 subsequent isolates), of other coronaviruses (CoVs) and of other (+)ssRNA viruses were investigated for the presence of such structures using the G4-iM Grinder (GiG) algorithm with some modifications. The quadruplex sequence structures identified were evaluated for the probability of occurring in infected cells, their position in the genome, and the degree of conservation in closely related viruses of the Coronaviridae family. The ‘best’ candidates for potential quadruplex formation were explored for stability by biophysical techniques (NMR, CD spectroscopy). Several relatively stable quadruplexes were identified which may be considered as possible targets for the development of novel antivirals.
The data observed are interesting. However, their presentation is rather confusing:

- The Materials and Methods section frequently refers to the Suppl. Mat. component of the manuscript for information which should be in the main text;

We thank the reviewer for point this out. We have now moved the complete Materials and Methods section back to the manuscript to ease the flow of the manuscript.

- The modifications of the GiG method used are not properly explained;

We are sorry to read that part of our approach was not easy to understand. An additional explanation of the modifications of the GiG method used has now been included in this response to the reviewers and further clarified in the supplementary material, section 1.

- The ‘detailed analysis of the Results’ is moved into Suppl. Mat. which is rather confusing and also has led to some duplications in panels of figures in the main text and in Suppl. Mat. ;

The supplementary material has been reorganized. Duplicated panels have been deleted. Our initial idea moving the detailed analysis of the results to the supplementary material was to make the text lighter for readers, but we understand now that this can be confusing.

- The frequency of quadruplex structure candidates is very high at the lax quadruplex definition, but the relevance of such data in biological terms is unclear;

Please see the General Remarks section at the beginning of this document.

The PQSs found with the LAX configuration by the search engine have also been evaluated by the scoring systems. This score will determine their probability of forming or not G4s in vitro. The biological relevance of these structures is therefore not dependent on the configuration used by the algorithm, but instead by the Score threshold we decide to apply as a binary classificatory system, (and of course by the efficiency of the score algorithm itself). The location in the genome will also affect the biological relevance.

- The significance of differences in conservation of potential quadruplex structures is not clear;

The conservation analysis of the PQS and PiMS was expanded up until January 2021. It now includes 17312 sequences instead of the original 3317. The analysis was expanded to study PQS and PiMS variants found in other strains too. Also, differences between clades and lineages were studied regarding the amount of candidates found and their divergence from the reference genome. This was added in a paragraph in the main text in Results and Discussion – SARS-CoV-2; and in the corresponding part of section 3 of the supplementary material.

The conservation of the structures was calculated as {% = 100 * Nº of genomes with the sequences (as a perfect match) / Nº of genomes in total}. Hence, higher conservation rates means that the sequence can be found in more genomes. It may be difficult to understand when we are speaking of very high conservation rates where the standard deviation plus the mean exceeds 100 (for example, 99.89 % +- 1.20) because differences are not significant. Reviewer 2 suggests comparing the conservation rate to the mean sequence identity of all the sequences studied here (17312, mean sequence identity 99.83%). However this may be also difficult to interpret and comprehend, because we will be comparing how similar the genomes are ( as { 100 * (identical positions) / (aligned positions + internal gap positions)}), with the percentage of genomes with a perfect match of the sequence. For example, PQS mean conservation rate is 97.7 % and the mean sequence identity is 99.8 %, yet we cannot say that PQSs are less conserved overall than the rest of the genome sections because they do not measure the same parameter (or at least we understand that they don’t measure the same parameter).

What we did instead of comparing between means of PQSs in the genome, is focus in the sequence conservation with which we can compare individually between candidates. For example, a candidate with a conservation of 100 is preferable than one with 99, although other parameters may intervene in selecting the candidate here because the differences are not likely significant. However, between a candidate with a conservation of 100 and a candidate with 27, where the difference is much more significant, this may facilitate which candidate to evaluate in vitro (the one with conservation of 100). For the least conserved candidate we may have to study first if it has variants and if these are specific to any lineage or clade. This was what was done here for the candidates with higher scores.

- Text and Legend of Fig. 2 differ in the nomenclature of structures assessed;

The sequences tested in the biophysical experiments mentioned by name in the text have been named as the ex-Figure 2, now Figure 4 A. (CoVID-RNA.G4.1, CoVID-DNA.G4.2). All other sequences mentioned in the Supplementary material have been also renamed accordingly.

- The findings of this manuscript are claimed to ‘greatly expand the current knowledge regarding quadruplexes and the SARS-CoV-2 in particular [refs 66-68]’ (lines 320/321), but no details are given;

A new section at the end of the manuscript was added where we compare our results to the results found with other search engines. In this section, we give the details of why our results expand the current knowledge regarding PQS and PiMS in the SARS-CoV-2.

- No particular information is given on compounds which could potentially react with (and disturb) quadruplex structures, using them as therapeutic targets.

For this particular piece of work, G4 ligands were out of the scope of the article as this is only the first step on the search for therapeutic targets. The description/investigation of G4 ligands is the next step in this work. However, we have now included a section (page XXX) in which we have added a small description regarding the advances and uses of G4-ligands as potential antiviral treatments, including those for the potential treatment of SARS-CoV-2.

Numerous clarifications are requested, and the presentation of this potentially very important information should be improved.

Specific Comments
Line
1 Reconsider title, e.g., ‘Potential G- and C-quadruplex structures of SARS-CoV-2 as potential targets for the development of antivirals’, or similar. [Since no antiviral candidates are even mentioned, consider: ‘G- and C-quadruplex structures of SARS-CoV-2 and their potential biological relevance’]

The title has been changed to: “Potential G-quadruplexes and i-Motifs in the SARS-CoV-2”.

18 A short paragraph should introduce the topic.

A short paragraph was added (page XX) to introduce the topic.

28 … the entire realm… Please clarify. …. in common with other species… Please specify.

Added the clarification “in the entire viral realm”. Specified “with other viral species”.

32 … may be suitable targets for… Please clarify (see comment above).

Added “suitable therapeutic targets for…”

38 Consider citation of: Lau SKP, et al. Possible Bat Origin of Severe Acute Respiratory Syndrome Coronavirus 2. Emerg Infect Dis. 2020 Jul;26(7):1542-1547.
Andersen KG, et al. The proximal origin of SARS-CoV-2. Nat Med. 2020 Apr;26(4):450-452.

The requested references have been added.

45 … pathogenic bacteria and viruses…

Corrected the plural of virus as viruses.

47 Consider citation of:
Menachery VD, et al. A SARS-like cluster of circulating bat coronaviruses shows potential for human emergence. Nat Med. 2015 Dec;21(12):1508-13.
Peck KM, et al. Coronavirus Host Range Expansion and Middle East Respiratory Syndrome Coronavirus Emergence: Biochemical Mechanisms and Evolutionary Perspectives. Annu Rev Virol. 2015 Nov;2(1):95-117.

Added. Thank you.

57 … DNA or RNA [read RNA throughout the ms; ARN is the French abbreviation.]

This typo has been corrected throughout the manuscript and supporting information.

74 Consider reading: … great potential as targets for virus inhibition…

Modified from “iMs present great potential for targeting viruses” to “iMs are interesting potential therapeutic targets for viruses”.

77 Clarify the description of data from ref. [49].

In the original article of G4-iM Grinder [old ref 49], we analyzed the human genome with the predefined configuration and compared its PQS and PiMS densities to that of several other pathogenic bacteria, parasites and viruses. Here, we found that especially the rubella virus was extremely rich in these PiMS candidates, compared to the human reference genome. Other viruses such as the hepacivirus and measles were also found to be similar to the human counterpart when measured in density.

Correct the site of publication to: NAR Genomics and Bioinformatics, Volume 2, Issue 1, March 2020, lqz005, <https://doi.org/10.1093/nargab/lqz005>

Corrected in the references. Thank you.

78 Support the statement by refs.

It is the same ref as line 77. We have rephrased the sentence to make it more clear. “In the same study, other viruses such as the measles and hepacivirus C, presented potential iMs densities similar to the human genome.”

79 Rephrase sentence: … to the ongoing research efforts related to the COVID-19 pandemic by investigating SARS-CoV-2 for the presence of quadruplex structures…

Changed. Thank you

84 Rephrase sentence.

Rephrased. “These PQS and PiMS have been evaluated according to their potential to form quadruplex structures in vitro and localization within the genome. The presence of confirmed quadruplex-forming sequences and the candidates’ frequency, uniqueness and conservation rates between 3297 different SARS-CoV-2 clinical cases, were also analyzed.”

111 … presence of known-to-form quadruplex structures… Please clarify.

“Known-to-form quadruplex structures” has been changed to “confirmed quadruplex-forming sequences”. These are the structures already known to form G4 or i-Motifs in vitro and which are included in the GiG database. These are matched with the results to detect the ones that have already been verified to form *in vitro*.

133 and 158f. Please clarify what ‘positive’ and ‘negative’ mean in this context. Are these designations just used for presentational purposes?

PQS and PiMS are measured in the same scale, as adapted from G4-Hunter. This scale range is from -100 to 100. 0 is a midpoint, where quadruplex formation probability is very low. For PQSs, the more positive the score is, the more probable to form G4. For i-Motifs, the more negative the score is, the more probable to form i-Motifs. Figure 1, B depicts this scale, and the thresholds used. More information can be found in the General Remarks section.

154 Fig. 1A. Consider showing a quadruplex structure.

Modified image to show a quadruplex. Rephrased the caption accordingly.

172f This summarizing referral of the reader to Suppl. Mat. is confusing. In addition, in detail there are partial duplications in figures of Suppl. Mat. and the main text. It should be considered to transfer essential components of Suppl. Mat. into the main text (including relevant figures). See comments below.

The supplementary material has been completely redesigned and part of it has been included in the main text to facilitate the flow of the article.

208 to 213. The meaning of this text is not clear. The concluding sentence ‘Here they may play their biological role if formed’ is speculative and should be omitted as long as no hard data are available.

The sentence has been modified to be more clear. It now reads, “These candidates concentrate in the N, S and the orf1ab genes, and especially in the nsp 1 and 3 (for PQSs), and in the nsp 3, 4 and 12 (for PiMS). The orf3a, orf8 and UTR regions also presented these candidates. Other genes, such as orb7a and b, and orf10 were found totally void of them”.

“Here they may play their biological role if formed” was deleted.

220 This interpretation of Fig. 2A does not describe what is shown. Please adjust accordingly.

Images have been redone, as has the conservation section of the candidates found in SARS-CoV-2.

224 and 230. The citation of components of Suppl. Mat. is out of order and confusing.

The supplementary material has been redesigned to make it clearer and easier to follow.

247 Fig. 2, panel C. Explain how COVID DNA.iM-1 was obtained and what this means in context.
In the materials and methods, under NMR, a phrase was added to state how the iM were specifically obtained. In the results- in vitro confirmation section, we added that NMR was used to verify the iMs. The explanation of the structure in its DNA form and its comparison to the SARS version follows. However, the RNA versions of the PiMS did not form iM. We included the pH in Figure 4C (previously Fig 2C).

260f Clarify sentence.

Line 260 speaks about the fact that no sequences already confirmed in vitro in other reports in literature were found in the SARS-CoV-2. Finding these sequences are one of the unique features of G4-iM Grinder and can save the researcher having to confirm their formation in vitro to confirm the predictions made here. These sequences have been described previously several times before in the manuscript.

267 … quadruplexes may be common and… for viruses to ‘live’, thrive and adapt… This statement is highly speculative and should be considered for omission.

Deleted this sentence.

293 and 296. Clarify: … CoVID-RNA G4-1… CoVID-RNA G4-2… The interpretation of the latter structure is highly speculative and should be omitted.

The section describing the quadruplexes found was rewritten as suggested by the reviewer.

310 … the opposite but with the same effect might also be happening… Please spell out more clearly what you want to say.
Changed to “Similarly, the pairing of C with G may also impede the formation of the C-based structures”.

318f This paragraph should contain details of how the data presented here augment those of refs. [60, 61, 63, 66-68]. Furthermore, the potential of developing novel antivirals interfering with quadruplex structure formation, should be assessed in more detail.

A new section was added where we compare the different results found in the SARS-CoV-2. Here, we explain how our results complement the other studies already published. Also, a brief summary regarding G4-ligands was added at the end.

476 Correct ref. , see comment above.

Fixed

520 Qu X is last, not first author.

Fixed

Suppl. Mat.

Page

3 The new functions developed and their exact website locations should be mentioned under Methods in the main text.

The link to the R package and to the new functions has been added to the methods section. These functions are fully documented within the package, in the webpage (along with the way to use them and helpful vignettes) and are also described in the supplementary material. Adding them to the method sections may be tedious/hard for non bioinformaticians as the journal scope is general.

4 The subheadings of Suppl. Fig. 1 could be incorporated into Fig. 1, making Suppl. Fig. 1 redundant.

Figure 1 was redone to display what the text is talking about in the section, regarding the SARS-CoV-2 and its relationship with PQS and PiMS, with those that |score| higher than 20, and their location and conservations in the genomes. Suppl. Fig.1 was enhanced to help understand the new section in the supplementary material (section 1), that talks about the algorithm.

5 Essential information of ‘in silico methodology’ should be in the Methods section of the main text.

The full Methods has been transferred to the main text.

6 The procedure of NMR and circular dichroism experiments should be in the Methods section of the main text.

The full Methods has been transferred to the main text.

7 A condensed version of this text should be transferred to the Methods section of the main Text.
Paragraph 2, line 3. Omit ref [2].

Within the Method section, the reference to the GISAID database has been included. Now it reads “The search was then expanded to 17312 different SARS-CoV-2 viral genomes sequenced during the pandemic (from December-2019 to January-2021, by different laboratories worldwide and downloaded from the GISAID database …”

8f Suppl. Fig. 2 panel A duplicates Fig. 2 panel A. Suppl Fig. 2 panels B-D could remain in Suppl Mat.

Panel A has been eliminated, and the Figure captions of Figure 2 in Supplementary material renamed. Panels B-D have been transferred to image 1 of the main article to match the segment that speaks about the virus and the location of these structures within its genome.

11f Abbreviated versions of these data should be transferred to the main text.

The figures of the supplementary material have been redone as a general suggestion from the reviewers.

14f and Suppl. Fig. 4 could remain in Suppl. Mat. in a condensed form.

Supp figure 4 explained the quadruplex comparisons between the different *Coronaviridae* family members, where the low density of SARS-CoV-2 compared to other family members is appreciable. Also from the figure, it can be appreciated how the *Coronaviridae* has, at the same time, lower densities than other Group IV +RNA viruses, although no differences were seen between different viric groups. We believe that this image is useful to situate the SARS-CoV-2 inside the virus realm and thus we have decided to keep it.

18f Biophysical experiments. The Suppl. Fig. 5 duplicates data of Fig. 2. The section should be transferred to the main text and abandoned in Suppl. Mat.

Figure 5 from supplementary material has been removed. Section A, has been included as last panel of figure 2 in the main manuscript. Here, we speak of the positive results, and in Figure 4 of the supplementary material we display the negative results of the experiments tested.

21f ‘Other bioinformatics figures’. Suppl. Figures 6-11 and 13-16 are not considered to be essential. Suppl. Figs. 12 and 17 could be considered for the main text.

Figures 6, 7, 8, 9,10 and 11 were deleted. Figure 12 was left in the supplementary material as Fig 6. Figure 13, 14 and 15 were also deleted. Figure 16 was left as Figure 7 because it states the number of G4s and i-Motifs that have been already confirmed to form in vitro in the genomes of each group of the virus kingdom. These are evidences that these structures can be found everywhere in the realm even with the information we already have in literature.

33f ‘Other biophysical figures’. Suppl. Figs. 18 and 19 could be considered for Suppl. Mat., preferentially Suppl. Fig. 19.

This section was merged into a single Figure. Figure 5. It was added after the biophysical description. It displays the negative results obtained in the biophysical results. The section ‘Other biophysical figures’ was eliminated.

35 to 37. Condensed components of this information (including relevant additional refs.) should be transferred to Methods of the main text.

The section was updated to include the new data obtained from the analysis of the 17312 SARS-CoV-2 viruses.

Similar information to that of the section can be retrieved from the package GitHub page, which however is more complete as we are continuously updating the page with the new results published in literature regarding the SARS-CoV-2. In the Methods section, a link to the data section was added. However, adding more information regarding the structure of the data to download and work with, may be too specific and of little interest in the main text.

Reviewer #2: In their manuscript the authors present a genome wide screen for putative
G- and C Quadruplexes sites (PQS and PiMS, respectively) in SARS-CoV-2 and other
viral genomes. For that purpose, the authors updated the pre-existing
R-based scanning tool G4-iM Grinder.
The genome wide screen in the SARS-CoV-2 reference genome resulted in about
300 PQS and about 200 PiMS. However, when the resulting scores were filtered
by a previously suggested value of |score| > 40, none of the predicted sites
remained. Therefore, the authors concentrated on those results with |score| > 20
which consisted of 71 PQS and 32 PiMS. The authors then compared these
sites to other viral genomes within the Coronaviridae, the entire group IV
and a selection of all virus genomes to find sites conserved within different
virus species. Furthermore, the authors conducted NMR and CD experiments on
a handful of selected high-scoring PWS and PiMS found in SARS-CoV-2 reference
genome. Here they found that both of the PQS tested do form a G-quadruplex.
The single PiMS, however, did not show formation of a C-quadruplex. However,
for the latter the authors also tested a DNA variant instead and found, that
in DNA this sequence indeed adopts a C-quadruplex conformation. Interestingly,
a single point mutation in the first loop region this DNA variant disrupts
the ability to form the C-quadruplex.

Overall, the article is very well written, and the analysis performed by the
authors is comprehensive and sound. Nevertheless, there are some minor points
that require more attention or need to be described in more detail. Furthermore,
in some instances the authors draw conclusions that I can't follow or agree
with.


In particular, I have the following remarks and questions:

1. In the methods section, the authors state that in their analysis they chose 3 different configurations of G4-iM Grinder, the lax, the default, and the 'original folding rule'. However, all Results are only for the lax configuration. If I understand correctly, this was the only configuration that yielded any results for SARS-CoV-2. If none of the results presented in this study (and the corresponding supplement), please correct the Methods section accordingly.
Along with that, I wonder how the authors justify their threshold of |score|>20. From the original G4-iM Grinder publication I took that |score|>40 is suggested for good prediction performance. Here, I am missing some mroe discussion about this lower threshold, especially in combination with the lax configuration, as I assume that this might greatly increase the number of false positive predictions!

The analysis with the predefined set, and the folding rule was added to compare the results with those of the lax configuration, and to be able to justify why we used the lax configuration. This was because, as the reviewer says, the lax configuration was the only one to allow G-runs of size two, which are fundamental to find the G-quadruplexes of the virus. More information can be seen in the General Remarks section, including about the score criteria. Section 1 of the supplementary material has been modified to briefly describe the search engine and scoring system.

We selected three different configurations for the analysis with G4-iM Grinder. The actual configuration and the results for each configuration for the SARS-CoV-2 can be found in Figure 1, B tables 1 and 2.

2. The authors should consider comparing their result of 73 PQS to those found
in another previous study by Ji et al. 2020 "Discovery of G-quadruplex-forming
sequences in SARS-CoV-2", Briefings in Bioinformatics (<https://doi.org/10.1093/bib/bbaa114>)

A paragraph was added comparing the results of different quadruplex algorithms on the virus (including that referenced by the reviewer, which used QGRS mapper for the analysis).

3. 3rd paragraph of the Materials and Methods section, line 125ff. The authors state that a more flexible configuration requires 'more computing power'. Maybe, I oversaw this but I wonder why this is the case and how the computation time scales with the choice of the different 'flexibility' parameters. Does the analysis scale linearly, quadratically, or even exponential in the number of
candidate sites? One can only speculate at this point. Especially, since the underlying scoring schemes (cGcC, G4Hunter, etc) seem to be independent on the flexibility of the sequence constraint.

Several parameter configurations were tested for G4-iM Grinder, to explore how different variables affect the performance time, disk size of results (bytes), the number of PQS found and the PQS found after applying the score filter of 40 and 20. The Genome of *S. cerevisaceae* was used because of its size (5 million bp) which is sufficiently big to do a representative analysis. G4-iM Grinder was run with different combinations of variables exploring how the G-run size, Loop size range, the number of bulges accepted per run and the total size of the PQS found affect these parameters. The conditions with BulgeSize = 2 and G-run minimal size = 2, were eliminated. In total 72 experiments were studied. The experiments were run on an intel core i7 on a single computer core.

G-run minimal size:	{2,	3,	4}

Loop size range:	{20,	30,	40}

Bulge Size:	{0,	1,	2}

MaxPQSSize:	{30,	40,	50}

The analysis on time, disk and results was,

The most influencial parameter on the responses time, disk and number of results (independently of score) is the Minimal Run Size, and especially between two and {three & four} minimal Gs (this allows GG to be detected as G-runs, instead of the classical GGG; or if BulgeSize = 1, GXG, where X is any nucleotide except G). BulgeSize also affects heavily, although in this analysis it was partially coerced by the MaxIL parameter, which was fixed. The Max loop size was significant for the time and size of the data, but not for the number of results. By the contrary, the total PQS size requirements (MaxPQSSize) was only found mildly significant for the number of results (especially between 30 and 50 max sizes), but not for the calculus itself. Simple linear models of the conditions were calculated, and results showed a rather poor fitting. When quadratic models were tested fittings did get better (data not show, R2 Adj, 0.6-0.9). Also interactions between the conditions have been seen to be significant. Thank you for the idea of the exponential models.

With this data, we also predicted how much time and resources would be required for the analysis of the human genome with G4-iM Grinder using the LAX configuration, using the genome of *S. cerevisiae* as reference. We extrapolated that it would take in between 310 and 851 h (12 – 22 days) for the analysis to complete using 7 cores of the i7 processors (out of 8), and take up to 173 gb of disk. RAM demands would be the most important limitation for the analysis.

***4.*** *Results and discussion SARS-CoV-2, 5th paragraph, lines 242-245, as well as Supplement Section 3, 'SARS-CoV-2, the Virus Realm and quadruplexes'. The authors matched PQS and PiMS found in SARS-CoV-2 in other viruses and claim that these findings 'cannot be explained by chance'. I argue against that, since there is no obvious relation between dsDNA viruses and the ss+virus SARS-CoV-2. Given the probability in the order of 1e-13 as derived in the
supplement and the vast size of the entirety of viral genomes, I would expect a
few sequences harboring common subsequences in the size of the PQS tested.
Note, that most viruses mutate much faster than bacteria, eucaryotes, or archaea,
so chances are, that subsequences of length of about 30 develop independently.
Especially if the other hypothesis is that dsDNA and +strand ssRNA viruses are
somehow related and have conserved these small pieces of subsequence during
evolution.*

With the statement of *'cannot be explained by chance',* our intention was not to imply anything about the origin of the sequences, or relationships between viruses. We just stated that this matches cannot be explained by chance taking into account the amount of sequences analyzed. Therefore, these matches make these PQS more interesting for a posterior analysis. For example, comparing their annotation files, if they repeat more times within the genome, if they have known-to-form G4 sequences within, or directly select it for *in vitro* confirmation. *'cannot be explained by chance',* was changed for *'cannot be explained by the number of sequences analysed',* to make it clearer. In addition, the sequence may also be important itself, like the telomere quadruplex sequence [GGG(TTAGGG)3] which is found in the human genome besides other animals and even parasites.

The matches in the analysis are sequences that match completely, or that the SARS-CoV-2 Sequence can be found within the comparing results. However the match has to be perfect. Hence in a genome where we can only have 4 options (G,C,T/U,A), the number of genomic combinations depends on its length as follows,

Combinations = 4^length of sequence^

For sequences of length 16, as the one matched for the SARS-CoV-2, will have 4^16^ combinatory possibilities. For one random match in the amount of combinations tested, the chance is 1/4^16^.

In the analysis of the virus realm, we analyzed nearly 215000 PQS and 215000 PiMS sequences with a length of 16 (which are the only ones that can have a possibility of being a match). Hence the possibility of finding a match in in these sequences due to randomness is

215000 * 1/4^16^ = 5e^-05^

Furthermore, finding a sequence with 3 random matches, such as the PiMS candidate found in common in the article, will have a possibility of,

(5e^-05^)^3^ = 1.25e^-13^

In both cases, the probability is insignificant.

Even if we consider all the sequences analyzed (independently of the length) as viable candidates for the match, the possibility due to randomness is of,

21.8 million * 1/4^16^ = 5.10e^-03^

Which is much lower than the typical 0.05 discriminatory alpha.

To complement the analysis, we randomly shuﬄed sequences (16 nucleotides long, with probability of G, C, U, A of 25%) to see if a match for a PQS or PiMS occurred. We tested 1 million and 10 million sequences, and in either case, no matches were obtained. The test was repeated 10 times for each condition. This is expected because the probability of 1 match here is of 4294 million: 1.

Additionally, we repeated the shuffling of nucleotides in sequences using as nucleotide probabilities the mean G, C, U, A values of the candidates found in the SARS-CoV-2 (0.352, 0.144, 0.266, 0.238). We tested 1 million and 10 million sequences, and in either case, no matches were obtained again. The test was repeated 10 times for each. We additionally tested 100 million sequences, an again no matches were found.

5. For the conservation of the PQS found in SARS-CoV-2 reference and the remaining
3297 SARS-CoV-2 genomes, please also state the overall sequence identity. Otherwise,
simply showing that the PQS are conserved by 98.6%+-7.4% renders it difficult to
assess whether this is expected, or unexpected.

Please see response to question 5 from reviewer 1 about conservation ranges for details. The segment about conservation was redone and improved.

The overall identity was added (99.83 %) although it is not clear how to compare both of the parameters.

6. Suppl. Figure 17 and related text. The examples given do not seem well chosen.
First, the linker sequences between the G-runs are quite large compared to the number
of layers (2). So I'd assume that, if at all, the resulting quadruplexes would be
exceptionally weak. Second, the regions of Cluster 1 and Cluster 2 are located
in the 5'UTR which is know to be well structured and conserved throughout all
betacoronavirus and even the remaining coronaviruses. In particular, Cluster 2
overlaps the well annotated SL5C which is required for replication. Cluster 2
resides in a highly complementary region that is known to form well conserved
secondary structure. This might also explain the low conservation of 27% among the
coronaviruses, if I'm not mistaken, since secondary structure, not G-quadruplex
seems to play the most important role here. The authors should therefore relate
their findings to known annotation of SARS-CoV-2.
Along with that, the authors should add to their discussion a paragraph about
the reliability of their predictions, if such can be assessed. Especially
the PQS (and PiMS) with just 2 layers and/or bulges are known to be less
stable, thus potentially do not form at all (in vitro or in vivo). Moreover,
even when NMR and CD measurements of the PQS suggest their formation, they
still have to compete against regular secondary structure formation when they
reside in their viral sequence context. The authors should elaborate on that
problem, at least in the discussion.

The PQSs and PiMSs in the clusters in 5’UTR region were selected because of the extremely low conservation rates (in the 3300 sequences, 27 %; in the 17000 genomes analysis, 9 %). With this figure we wanted to see and explore what was happening here, despite the previous knowledge regarding the area for other Coronaviridae family members ([10.1016/j.virusres.2015.02.025](https://dx.doi.org/10.1016%2Fj.virusres.2015.02.025)). However, given the low scores of the PQS and PiMS in the area, most likely these structures are not able to form quadruplex structures, which may be explained by the presence of other secondary structures that makes quadruplex structures superfluous, as commented by the reviewer.

Hence, we have deleted the image.

The reliability of the predictions are based on G4Hunter and PQSfinder. A new section was added in the supplementary material which explains the evaluation systems. PQSfinder takes into account the length of the tracks as well as the number of bulges in its classification process. G4Hunter takes an indirect route by evaluating the G richness and C skewness. When explaining the evaluating scores, the fact that they still have to compete against regular secondary structure formation when in their viral sequence context has been addressed.

“Hence, the higher the absolute value of the score, the more probable these are capable of competing against other regular secondary structures for formation, and the more probable they are to give rise to a G4 (if its positive) or an i-Motif (if its negative).” Was added in the corresponding section.

General remarks:

- There are multiple occurrences of ANR instead of RNA throughout the manuscript
and Supplement. Please correct them.

Changed ARN to RNA in row 57. Changed ARN to RNA in Supplementary material page 3. Section 1, A line 4.

- In the 4th paragraph of the introduction, there is a 'nucleic acid sequences'
right after DNA and RNA. This is redundant, since the NA in both already stands
for Nucleic Acid

Removed 'nucleic acid sequences'

- In the second paragraph of Materials and Methods, line 112. What do the
authors attempt to convey with 'presence of known-to-form quadruplex structures
sequences'? Maybe a simple 'presence of known-to-form quadruplexes' would suffice?

It would. Thank you. Changed to quadruplexes instead of quadruplex structures sequences.

**Reviewer #3:** Thank you for the opportunity to review the manuscript: “Exploring G- and C-quadruplex structures as potential targets for the severe acute respiratory syndrome coronavirus 2". This manuscript is presenting analyses of potential G-quadruplex-forming sequences (PQS) in the genome of SARS-CoV-2 and related viruses. It was interesting to read this, but there are several points which must be improved remarkably before its publication. The main problems are

1. Conclusions of the analyses are wrongly interpreted,

2. The various methods must be used for their results evaluation and

3. The used methods must be described in manuscript with details. Although I found this manuscript interesting the major revision is necessary before its publication. I recommend a major revision of this manuscript.

Major points:
1. Conclusion are misleading. The authors find that compare to other tested viruses, there are no potential G-quadruplex forming (PQS) sequences in SARS-cov2 genome. Then they changed the algorithm for PQS search – “lax” configuration – with loop size 20bp – and claimed that these PQS could be may be suitable targets. This approach is wrong. The stability of these G-quadruplexes will be extremely low, so their formation in vivo is very doubtful. The right conclusion will be that compare to other genomes – the G-quadruplexes are probably very rare (if presented) in SARS-cov2.

Please see General Remarks section.

We understand the concerns of the reviewer regading the potential stability or lack of thereof of the identified sequences. The configurations for the algorithm allows for the detection of the sequences that fit that pattern. The more lax, the more sequences you detect. However, they all have to be evaluated by the scoring systems. PQSfinder scoring system for example, considers directly the loop sizes in its evaluation of the sequence’s probability of formation. G4Hunter does it indirectly through the quantification of the opposite nucleotide; although it “ignores” other nucleotides (T/U, A)

QGRS mapper predefined configuration is to accept loops between 0 and 36 bases long, which is double that of the lax configuration. Still, the search with QGRS mapper only yields 25 results. This is because the loop size explains very little variance as explained in Answer 4 to Reviewer 2.

2. The alternative algorithms must be used as a control and discussed (for example QGRS Mapper and G4Hunter web are freely available and easily accessible for quick evaluations).

Please see the General Remarks section.

A paragraph was added at the end of the discussion where we have analyzed the results with different algorithms already published.

3. The Title should be improved, the authors did not target any structures, just predicted them.

Changed to : “Potential G-quadruplexes and i-Motifs in the SARS-CoV-2”

4. Description of experimental methods in Material and Method section is missing.

It description has now been included in the main manuscript.

5. How the PQS score is set? What these number means? Please compare with G4Hunter score and QGRS score.

Please see General Remarks section.

A paragraph was added at the end of the discussion. Here we analyzed the results obtained with different algorithms already published.

A description to the Score system was introduced in the first section of the supplementary material.

6. The comparison of PQS in real genomes must be compared statistically with scrambled/random sequences – it is possible than the same amount or maybe more PQS will be in scrambled sequence. With this control your analyses do not prove anything.

The probability of the matches being to randomness is demonstrated in the answer to reviewer 2, issue 4. If by scrambled the reviewer means overlapping, G4-iM Grinder detects all overlapping sequences (with method 2, which is the one used in the manuscript; size dependent overlapping search method). Hence, all combinations of overlapping sequence are retrieved and compared in the match.

7. Do not use suggestion the the Result part. Just described your results (e.g. line 231: Here they may play their biological role if formed., line: 266, 277: „may be“. We are not interested what „may be“ in Results, but we would like to see the facts only. Keep your theories and “maybe” into Discussion part.

The manuscript has the result and discussion sections joined as the main part of the article. This is because we instead have divided the results and discussion into, a) SARS-CoV-2; b) SARS-CoV-2 and the Coronaviridae family; c) SARS-CoV-2 and the virus realm. Hence, each part has its result and is followed by the discussion. This is why our theories and maybes follow the results analysis. We think that separating as:
I) Results	a) SARS-CoV-2;	b) SARS-CoV-2 and the Coronaviridae family;	c) SARS-CoV-2 and the virus realm
II) Discussion	a) SARS-CoV-2;	b) SARS-CoV-2 and the Coronaviridae family;	c) SARS-CoV-2 and the virus realm

would complicate linking the results to the discussion as the reader may more easily get lost between comparisons between groups.

8. Figure 2B. Compare results with already in vivo proved sequences.

A new section was added where we compare the results with other reported sequences. Figure 2B (now 4B) is one of them, which since then was confirmed in vivo. The G4 topology in both cases is the same as it is the same G4. Additionally, a phrase was added in the manuscript to further point this out “In fact, it has been shown in a recent study that a known G4-ligand can interact with this sequence and reduce the expression of the N protein [65]” within the in vitro candidate confirmation section.

9. Figure 2C, Show the same result with confirmed iM and compare the spectra.

The authors do not understand this request from the reviewer. Figure 2C (now Figure 4C), is the NMR spectra of the PiMS sequence in its DNA version at different pH, that confirms that the sequence forms an iM. It is a confirmed iM and hence instead of comparing it with another confirmed iM, the comparisons were made between the DNA (which forms iM) and its RNA versions (which does not form iM), and between the CoVID iM and the SARS version of the iM, which with only one variation is not able to form in vitro an any pH tested. The spectra of the negative results are in the supplementary material, Figure 5. A reference to the Figure in the supplementary material was added in the main text.

Minor points:
1. Please polish the terminology. I-motifs are formed by four strands, however to use C-quadruplex is unusual, because the structure is completely different from G-quadruplexes. Fours strand structures: G-quadruplexes and i-motifs.

The term C-quadruplex has been changed to i-Motif.

2. Please do not repeat the same first sentence in Methods and Results section.

Changed the results first sentence. “The genome of the SARS-CoV-2, and that of many other viruses, were analysed with G4-iM Grinder in search off potential quadruplex (both G4 and iM) therapeutic targets.”

3. Line 195 and 196 – do not discuss in Result section.

Please see answer 7.

4. Line 294 and 295 – again this is not your result so move to discussion part

Please see answer 7.

5. Line 316 and 317 – I do not understand. How your results on RNA viruses proved “the especially for DNA, G4-iM Grinder can be used”? Any algorithm can be used if you used “lax” settings.

Please see General Remarks section.

Our results here, (which consisted of the analysis of over 17000 SARS-CoV-2 genomes, and over 6600 genomes of other viruses), included both DNA and RNA genome types.

The lax configuration can only be used in algorithms that allow modification of these parameters. Most don’t, for example QGRS mapper does not allow bulges in runs; G4Hunter does not allow any search engine configuration besides the window size and does not define the limits of the PQSs. Hence, most cannot find the results we can found with G4-iM Grinder.

However, all the results must be evaluated by the scoring systems to see which have a possibility of forming. G4-iM Grinder uses the mean of G4-Hunter and PQSfinder for this.

The line the reviewer is referring speaks concretely about i-Motifs and DNA. This is because very few other algorithms have the ability to detect them, and only one besides G4-iM Grinder (G4Hunter) has the ability to evaluate them (G4-iM Grinder uses also an adaptation that allows PQSfinder to punctuate i-Motifs; and the system was tested on over 100 DNA i-Motifs described in literature). On the contrary, RNA i-Motifs have been very scarcely studied because of their reduced stability compared to their DNA counterparts, and hence it’s not currently possible to do efficient prediction model for them. However, the specific line was deleted because as suggested by the reviewer.
