# Supplementary material for: Potential G-quadruplexes and i-Motifs in the SARS-CoV-2
Source: PLoS One. 2021 Jun 8;16(6):e0250654. doi: 10.1371/journal.pone.0250654 (PMC8186786; doi:10.1371/journal.pone.0250654)
Supplement: Attachment — Submitted filename: Reviewer.answers.2.March.docx [file pone.0250654.s003.docx]

Dear Reviewers and Editors,

Thank you again for your comments and suggestions.

Reviewer #2: The authors revised their manuscript and properly addressed all the comments and questions raised in my previous report.

Thank you.

Reviewer #3:

1. The experiment with the "shuffled" or "random" sequence was preformed wrongly. : "To complement the analysis, we randomly shuffled sequences (16 nucleotides long)."
   Please take complete virus sequence then shuffle and analyse this 29903 nt long sequence - and compare the number of PQS sequences in the real virus sequence and "shuffled" sequence.

What the reviewer suggests to do is not what we intended to proof. The reviewer suggests to shuffle the genome of the SARS-CoV-2 and compare the results of the random shuffle with the actual results of the virus, as to determine if the number of sequences found in the virus are significant or just due to randomness.

However, this was not our intention because either by randomness or not, the sequences found in the virus are potential quadruplex structures which are interesting therapeutic targets once confirmed they form *in vitro.* Then we can evaluate their potential to be targeted *in vivo*. Several authors actually describe quadruplexes as ubiquous structures of the genome, expected in rich and long enough genomes. However, not only these parameters are important, as also how the genome is organized is critical. For example, two different genomes composed of 50 % G could be:

1. **G**C**G**C**G**C**G**C**G**C**G**C**G**C**G**C**G**C**G**C**G**C**G**C
2. **GGG**CCC**GGG**CCC**GGG**CCC**GGG**CCC

Which gives very different results in terms of G4-forming capabilities.

What we did in the manuscript (and we rebutted earlier in the previous revision submission) is testing if the matches of potential candidates found in the SARS-CoV-2 genome and found also in common with three OTHER VIRUSES (hence they match) are significant or, by the contrary, if they are the results of randomness due the number of sequences analyzed (over 2 million sequences analyzed here in total).
For the calculus and confirmation of this point, please view the previous rebuttal, response 4 to reviewer 2 (please note that the test here was done with shuffled 16 long sequences, as well as later, with all the sequences found, independently of size).

Despite this fact, we thank the reviewer for the suggestion.
We did as the reviewer asked and calculated, on a shuffled 29903 long sequence, the nº of PQS candidates found with the same LAX configuration.
We did this 500 times with the nucleotide composition of the SARS-CoV-2 (G= 0.196, C= 0.184, U= 0.266, A = 0.238).

The results were:

|  | Total PQS | PQS Score >= 20 | PQS Score >= 40 |
| --- | --- | --- | --- |
| Shuffled MEAN ± SD | 362.6 ± 42.4 | 96.7 ± 21.7 | 3.0 ± 2.6 |
| REAL  SARS-CoV-2 PQSs | 323 | 71 | 0 |
| P (greater) | < ${1\times10}^{-16}$ | < ${1\times10}^{-16}$ | < ${1\times10}^{-16}$ |
| significance | *** | *** | *** |

Were p is the t-test’s p comparing the mean shuffled results with the actual real results, as to determine if the shuffled mean is equal or greater than the real values.

**Figure X1**, PQS found per filtering criteria for 500 random shufflings of 29903 nucleotides with the genomic proportions of the SARS-CoV-2 (G= 0.196, C= 0.184, U= 0.266, A = 0.238).
In red, the number of PQSs found for the SARS-CoV-2 genome.

As it can be seen, the number of PQSs in the SARS-CoV-2 is in the lower end of the distribution of possible candidates with the same length and composition. This is the effect of the nucleotide organization that restricts the number of candidates in the genome.

Similarly the results are even more different for PiMS.

Here, the results were:

|  | Total PiMS | PiMS Score <= 20 | PiMS Score <= 40 |
| --- | --- | --- | --- |
| Shuffled MEAN ± SD | 250.3 ± 32.7 | 60.3 ± 16.1 | 1.5 ± 1.6 |
| REAL  SARS-CoV-2 PiMSs | 189 | 32 | 0 |
| p | < ${1\times10}^{-16}$ | < ${1\times10}^{-16}$ | < ${1\times10}^{-16}$ |
| significance | *** | *** | *** |

Figure X2, PiMS found per filtering criteria for 500 random shufflings of 29903 nucleotides with the genomic proportions of the SARS-CoV-2 (G= 0.196, C= 0.184, U= 0.266, A = 0.238).
In red, the number of PiMS found for the SARS-CoV-2 genome.

This furthers corroborates our findings that indicate the low quadruplex prevalence in its genome, which has been mentioned in the article several times.

The scarcity of these structures can potentially be related to its use as regulatory elements of the genome.

The end of the section SARS-CoV-2 and the virus Realm was modified to include a small segment about this. It now reads like:

“Altogether, the SARS-CoV-2 genome displayed a quadruplex candidate scarcity when compared in a macroscopic perspective to the virus realm. Its PQS and PiMS densities were in the lower end of results from the *Coronaviridae* family, which itself was in the lower end of the (+) ssRNA Group IV (in an approximate ratio of 1:2:4 for PQS and 1:2:8 for PiMS). When put into the entire virus realm context, the SARS-CoV-2 PQS density was lower than 5813 other viruses analysed (out of 6680), whilst PiMS density was lower than 6125. Furthermore, when we compared the SARS-CoV-2 reference genome results with the results of five hundred randomly shuffled genomic sequences of size and composition equal to that of SARS-CoV-2, the number of candidates found in the SARS-CoV-2 was significantly lower than the mean expected number of candidates for the genome’s size and composition. Whilst 362 ± 42 PQSs were expected, only 323 were found in the SARS-CoV-2. Similarly, 97 ± 22 PQSs that score over 20 and 3.0 ± 2.6 candidates that score over 40 were expected with this genomic size and composition, whilst 71 and 0 were found in the virus, respectively. For PiMS the total number of expected candidates for the SARS-CoV-2 size and genome composition was of 250 ± 33, whilst candidates that score -20 or less and -40 or less was of 60 ± 16 and 1.5 ± 1.6, respectively. However, SARS-CoV-2 presented only 189, 32 and 0 PiMSs for each of these respective groups. Although the SARS-CoV-2 genomic organization limits the number of potential quadruplex structures almost to its minimum, other viruses with similar low quadruplex densities were identified here to possess confirmed G4 and iM sequences within, supporting the potential these structures have for targeting the SARS-CoV-2.”

2. The abstract do not correspond to the results. It was found that there are less PQS in Coronaviridae compare another viruses. This fact must be part of the abstract and disscussed accordingly.

This was not commented or suggested within the first review.

In the abstract, we mention that we have compared the SARS-CoV-2 quadruplex results with other Coronaviridae family members, and the entire virus realm. Given the small space given for the abstract, we mention only the most important and relevant results, which are that we have identified with G4-iM Grinder a set of 512 potential sequences in the SARS-CoV-2, and that the most interesting of them were confirmed in vitro. Both of these statements mentioned in the abstract correspond to the results we have enclosed in the manuscript.

The actual Coronaviridae family quadruplex scarcity, when compared to the rest of the virus realm, is not addressed in the abstract because although interesting, these results are less important than those previously mentioned. Furthermore, the manuscript is primarily focused on the SARS-CoV-2.
In the manuscript, we discuss in detail the differences between the SARS-CoV-2, the *Coronaviridae* family members and other virus families and groups.

3. "hundreds of potential quadruplex candidates were discovered" - please use exact numbers and significance in the abstract statements.

We have changed the phrase to “512 potential quadruplex candidates were discovered”.
